# Outdoor‐Useable, Wireless/Battery‐Free Patch‐Type Tissue Oximeter with Radiative Cooling

**DOI:** 10.1002/advs.202004885

**Published:** 2021-03-09

**Authors:** Min Hyung Kang, Gil Ju Lee, Joong Hoon Lee, Min Seok Kim, Zheng Yan, Jae‐Woong Jeong, Kyung‐In Jang, Young Min Song

**Affiliations:** ^1^ School of Electrical Engineering and Computer Science (EECS) Gwangju Institute of Science and Technology (GIST) 123, Cheomdangwagi‐ro, Bukgu Gwangju 61005 Republic of Korea; ^2^ Department of Biomedical Biological and Chemical Engineering University of Missouri Columbia MO 65211 USA; ^3^ Department of Mechanical and Aerospace Engineering University of Missouri Columbia MO 65211 USA; ^4^ School of Electrical Engineering Korea Advanced Institute of Science and Technology (KAIST) 291 Daehak‐ro, Yuseong‐gu Daejeon 34141 Republic of Korea; ^5^ Department of Robotics Engineering Daegu Gyeongbuk Institute of Science and Technology (DGIST) Daegu 42988 Republic of Korea; ^6^ Anti‐Viral Research Center Gwangju Institute of Science and Technology (GIST) 123, Cheomdangwagi‐ro, Bukgu Gwangju 61005 Republic of Korea; ^7^ AI Graduate School Gwangju Institute of Science and Technology (GIST) 123, Cheomdangwagi‐ro, Bukgu Gwangju 61005 Republic of Korea

**Keywords:** daytime radiative cooling, nonmetallic/flexible radiative cooler, outdoor useable oximeter, thermal management, wearable optoelectronics

## Abstract

For wearable electronics/optoelectronics, thermal management should be provided for accurate signal acquisition as well as thermal comfort. However, outdoor solar energy gain has restricted the efficiency of some wearable devices like oximeters. Herein, wireless/battery‐free and thermally regulated patch‐type tissue oximeter (PTO) with radiative cooling structures are presented, which can measure tissue oxygenation under sunlight in reliable manner and will benefit athlete training. To maximize the radiative cooling performance, a nano/microvoids polymer (NMVP) is introduced by combining two perforated polymers to both reduce sunlight absorption and maximize thermal radiation. The optimized NMVP exhibits sub‐ambient cooling of 6 °C in daytime under various conditions such as scattered/overcast clouds, high humidity, and clear weather. The NMVP‐integrated PTO enables maintaining temperature within ≈1 °C on the skin under sunlight relative to indoor measurement, whereas the normally used, black encapsulated PTO shows over 40 °C owing to solar absorption. The heated PTO exhibits an inaccurate tissue oxygen saturation (StO_2_) value of ≈67% compared with StO_2_ in a normal state (i.e., ≈80%). However, the thermally protected PTO presents reliable StO_2_ of ≈80%. This successful demonstration provides a feasible strategy of thermal management in wearable devices for outdoor applications.

## Graphical abstract

This article presents a radiative cooled wireless/battery‐free patch type tissue oximeter with nano/microvoids polymer (NMVP) for eliminating the thermal issue of optoelectronics. The NMVP integrated tissue oximeter serves a temperature within ≈1 °C on the skin under direct sunlight relative to indoor measurement, delivering reliable tissue oxygen saturation, unlike normally black encapsulated devices.

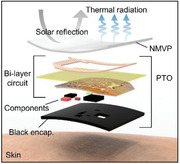

## 1 Introduction

Body‐worn electronic systems for health‐care applications have been developed with real‐time monitoring of various biological signals (e.g., sweat,^[^ ^1^ ^]^ humidity,^[^ ^2^, ^3^ ^]^ ultraviolet dosimeter,^[^ ^4^, ^5^ ^]^ oximeter,^[^ ^5^, ^6^, ^7^, ^8^, ^9^ ^]^ electrocardiac,^[^ ^9^, ^10^ ^]^ strain,^[^ ^10^, ^11^, ^12^ ^]^ tactile,^[^ ^13^ ^]^ stress^[^ ^14^ ^]^ sensors). Among them, the photoplethysmography (PPG) sensors (e.g., pulse oximeters and tissue oximeters) provide various biometric information from blood by penetrating light into skin: the pulsatile signal and tissue oxygenation can be measured using pulse and tissue oximeters.^[^ ^5^, ^6^, ^7^ ^]^ However, in the wearable configuration in close contact with the skin, heat generated by the light source is accumulated,^[^ ^15^, ^16^, ^17^ ^]^ which not only leads to inaccurate signal acquisition due to operation out of thermoneutral zone^[^ ^18^, ^19^, ^20^, ^21^, ^22^ ^]^ and deterioration in the light source performance^[^ ^23^, ^24^, ^25^, ^26^ ^]^ but can also burn the skin.^[^ ^16^ ^]^ Various approaches have been reported to release accumulated heat from PPG sensors, such as a thin metallic heat sink to dissipate the concentrated heat^[^ ^27^ ^]^ and a copper/paraffin bilayer acting as a heat sink based on phase variation.^[^ ^28^ ^]^ However, metallic layers hinder wireless communication between the receiver and transmitter^[^ ^29^, ^30^ ^]^ and phase shift materials function irreversibly at high temperatures of ≈50 °C.

In addition to the internal heat source (i.e., device self‐heating), the external heat source (i.e., the Sun) should be considered to exploit wearable devices outdoors. Conventional wearable oximeters reported in the literature have adopted a black encapsulation layer to block ambient light. Using the conventional layout in outdoor environments considerably exacerbates device heating owing to solar energy absorption. Previous works could not provide an obvious solution for these challenges. Table S1 in the Supporting Information lists the previous sensors and their thermal management methods in detail. Meanwhile, recently reported passive radiative coolers with high solar reflectivity and thermal radiation are suitable for not only the thermoregulation of PPG sensors but also the rejection of solar energy gain.^[^ ^31^, ^32^ ^]^ Fundamentally, passive radiative cooling is based on minimizing solar absorption and maximizing thermal radiation to outer space (≈3 K) through the long‐wave infrared (LWIR) atmospheric window (i.e., 8–13 µm wavelength).^[^ ^31^, ^32^ ^]^ State‐of‐the‐art radiative coolers have been based on metallic mirrors to minimize solar absorption, multilayered dielectrics on silver,^[^ ^33^, ^34^, ^35^ ^]^ and dielectric–polymer composites on silver.^[^ ^36^, ^37^, ^38^ ^]^ However, these designs also block the channel for wireless communication. Nonmetallic/flexible cooling structures are strongly demanded for thermal management of wearable and wireless optoelectronics. Although various approaches exhibit the design of nonmetallic radiative coolers (Table S2, Supporting Information), their designs were optimized for paintable coolers,^[^ ^39^ ^]^ radiative cooling wood,^[^ ^40^ ^]^ cooling/heating mode conversion,^[^ ^41^, ^42^ ^]^ and colored coolers.^[^ ^43^ ^]^ Fortunately, polymer–air composite radiative coolers have provided insight into user comfort from wearable PPG sensors.^[^ ^39^, ^40^, ^41^, ^42^, ^43^, ^44^ ^]^

Here, we introduce a new platform of wearable optoelectronics that is free from heat and light concerns, which enables assessing athletic performance in outdoor environments under direct sunlight. For compatibility with wearable devices, a nano/microvoids polymer (NMVP), a nonmetallic and flexible cooler, is realized using two perforated polymers (i.e., polymethylmetacrylate (PMMA) and styrene‐ethylene‐butylene‐styrene (SEBS)), which are optically and mechanically optimized and fabricated using a self‐assembly method.^[^ ^39^, ^44^ ^]^ The NMVP shows flexible features and exceptional optical characteristics, with near‐unity reflectivity and emissivity in the solar spectrum and atmospheric window, respectively. In addition, we design and fabricate wireless, battery‐free, and patch‐type tissue oximeter (PTO) with compact dimensions (20 × 17 × 2 mm^3^), which can monitor the oxygenation of muscle for athletic purposes. Integrating the NMVP with the PTO, we develop an outdoor‐useable tissue oximeter for assessing endurance performance under direct sunlight. We experimentally confirm that the NMVP‐integrated PTO obtains reliable measured results, both indoors and outdoors, whereas a conventional black elastomer (BE)‐ and white elastomer (WE)‐integrated PTO shows inaccurate tissue oxygenation results owing to thermal disturbance.

## 2 Result

**Figure 1:**
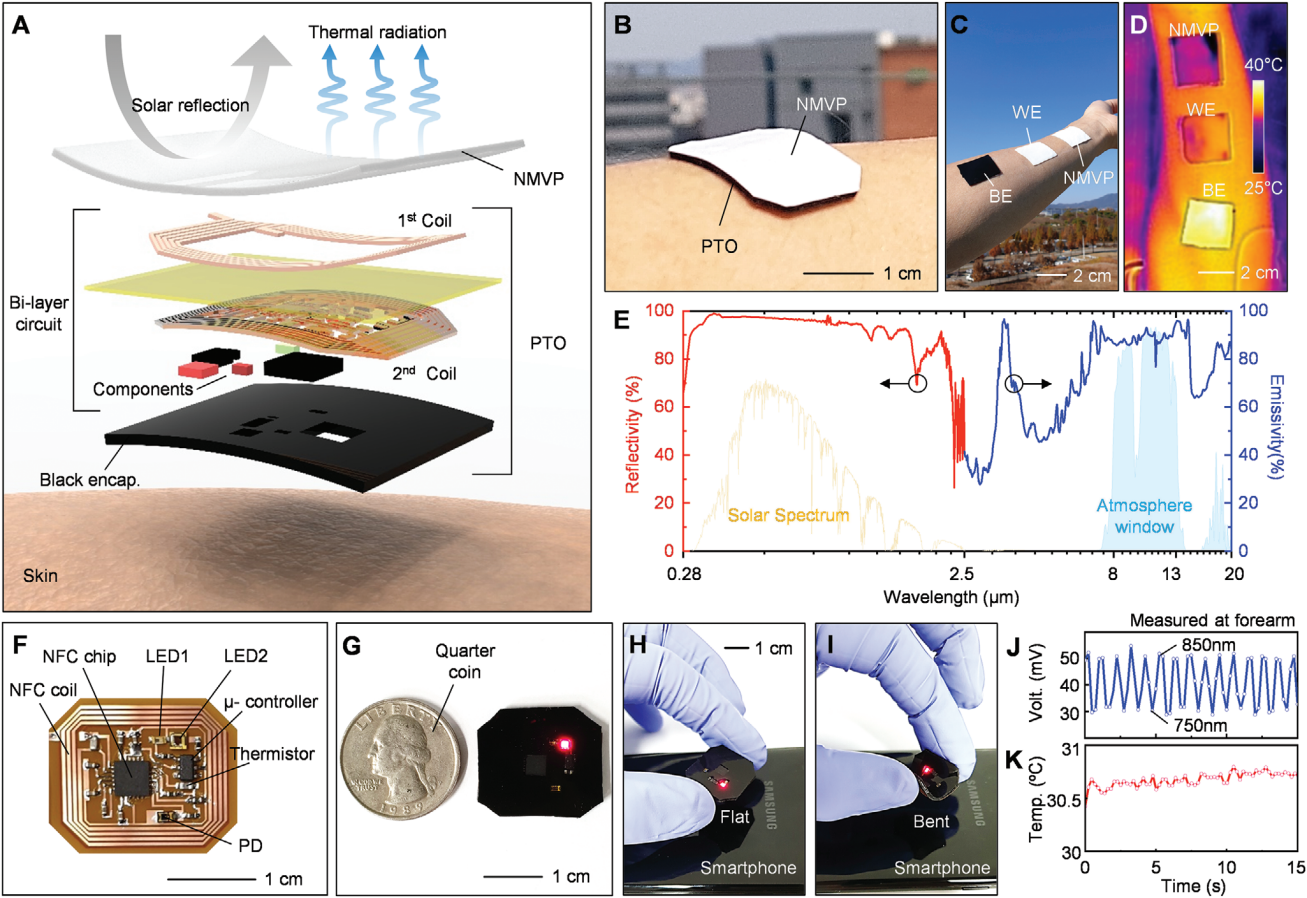
Wireless/battery‐free patch‐type tissue oximeter with radiative cooler. A) Exploded view of schematic illustration of the constituent layers. B) Photograph of the device mounted on forearm. C) Photograph and D) thermography of the samples such as black elastomer (BE), white elastomer (WE), and NMVP on human bodies. E) Reflectivity and emissivity spectra from the visible to far‐infrared wavelength range of NMVP. Optical images of F) unencapsulated device and G) encapsulated device. The wavelengths of LED1 and LED2 are 850 and 750 nm, respectively. Quarter coin highlights the miniaturization of the device. Photographs of the device during wireless operation with smartphone in H) the flat state and I) the bent state. J,K) Wirelessly obtained light and temperature data by photodetector and thermistor, simultaneously.

**Figure** **1**A illustrates an exploded view of the constituent layers of the NMVP‐integrated PTO, which is divided into three parts: 1) NMVP layer for radiative cooling and blocking external lights, 2) bilayer flexible circuits composed of passive/active electric components, and 3) black encapsulation layer to block the direct transmission of light from the light‐emitting diode (LED) to the photodiode (PD), and protect electrode and component. The NMVP is fabricated with a thickness of ≈500 µm in consideration of mechanical softness and cooling performance. In the circuit, a dual‐layer interconnect matrix with a 60 µm thick polyimide separation layer is formed for high wireless power transfer within a compact form. The bilayered near field communication (NFC) coil increases the efficiency of wireless power and data communication. Biocompatible double‐sided tape was used for adhesion with skin. See the device adhesion test in Figure S1 in the Supporting Information. Because each layer is flexible, the NVMP‐integrated PTO can be conformably contacted with the skin (Figure 1B).

As a result of comparing the temperature change of the NMVP, BE, and WE after operating for 8 min outdoors in the daytime, the NMVP maintained the indoor skin temperature whereas the temperature of the BE and WE rose to ≈40 and ≈35 °C, respectively (Figure 1C,D). Figure 1E shows the spectral characteristics of the NMVP, with high reflectivity within the solar spectrum and high emissivity in the atmospheric window (i.e., 8–13 µm). Therefore, the NMVP can effectively reflect sunlight to minimize solar absorption and radiate thermal energy through the atmospheric window to outer space, thereby achieving cooling effects. Figure 1F displays a photograph of an unencapsulated PTO, which is composed of an NFC coil and a circuit with soldered electric components (i.e., NFC chip, µ‐controller, LED, PD, thermistor, resistor, and capacitor). The distance between the LEDs and the PD, namely the interoptode distance, was set to 9 mm based on Monte Carlo ray tracing simulation. See Note S1 and Figure S2 in the Supporting Information for details of the simulation. The circuit operates based on the NFC system for wireless power transfer to achieve battery‐free operation, making the device compact. See Figure S3 in the Supporting Information for the detailed PTO operating system. Detailed PTO fabrication is explained in the Experimental Section.

As displayed in Figure 1G, a quarter coin is placed next to the encapsulated PTO to highlight the compact device size. Figure 1H,I demonstrates wireless operation of the PTO in both flat and bent geometries connected with smartphone. Figure 1J,K shows light and temperature data measured by the PTO simultaneously. See Videos S2 and S3 in the Supporting Information to confirm wireless operation of the PTO and data acquisition by connecting with smartphone using a customized android application.

**Figure 2:**
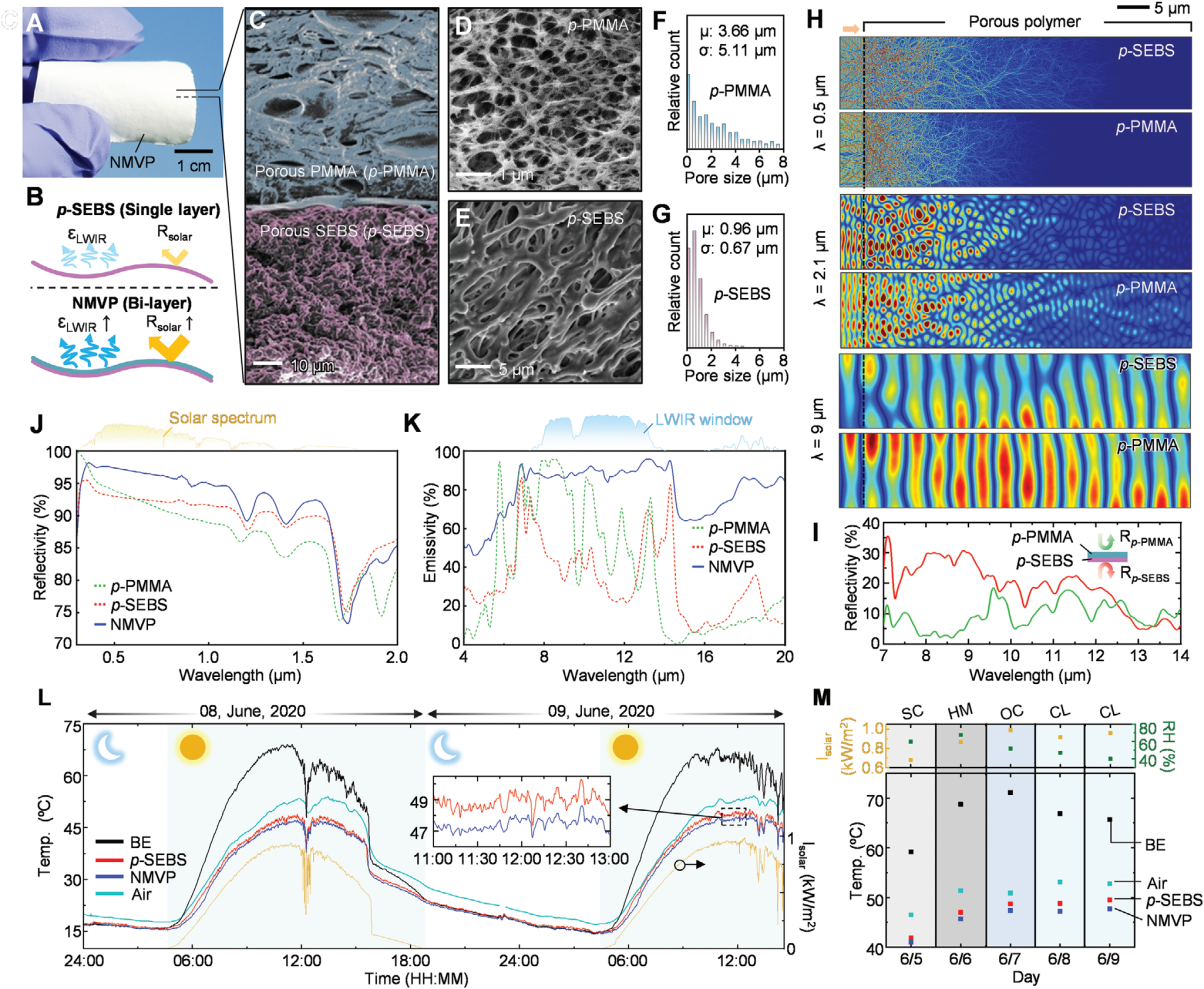
Structural, optical, and thermal characterizations of NMVP. A) Photograph of NMVP in bent state. B) Schematic illustration for improving solar reflection and LWIR emission owing to Mie scattering enhancement and antireflection effect of gradual refractive index. C) False‐colored scanning electron microscope (SEM) image of NVMP. Magnified SEM images of D) *p*‐PMMA and E) *p*‐SEBS. Pore size distributions of F) *p*‐PMMA and G) *p*‐SEBS. H) Electric field distributions for computational models of *p*‐PMMA and *p*‐SEBS at the wavelengths of (top) 0.5, (middle) 2.1, and (bottom) 9 µm. I) Measured reflectivity spectra of NMVP at the surfaces of *p*‐PMMA (green line) and *p*‐SEBS (red line). J) Reflectivity and K) emissivity of *p*‐SEBS, *p*‐PMMA, and NMVP. L) 38 h continuous temperature measurement of *p*‐SEBS, NMVP, and BE. Yellow line indicates the solar intensity, *I*_solar_. M) Measurement reliability of NMVP cooling ability by monitoring for several days. ‘SC’, ‘HM’, ‘OC’, and ‘CL’ refer to scattered, humid, overcast, and clear.

Using a phase‐inversion method, which is an affordable and self‐assembling method of production, the NMVP is implemented, and the fabrication is explained in the Experimental Section. **Figure** **2**A displays a photograph of the optimized NMVP. The NMVP is a bilayered radiative cooler, which is composed of porous PMMA (*p*‐PMMA) and porous SEBS (*p*‐SEBS). By coating the *p*‐PMMA layer on *p*‐SEBS, solar reflection (*R*_solar_) and thermal emission (*ε*_LWIR_) are enhanced due to Mie scattering enhancement within the solar spectrum and the antireflection effect within the LWIR window (Figure 2B). Figure 2C shows a scanning electron microscopy (SEM) image of the fabricated NMVP composed of *p‐*PMMA and *p*‐SEBS. Each polymer layer exhibits different porous morphologies and the magnified SEM images explicitly reveal the structures (Figure 2D,E). The *p*‐SEBS has a narrow range of pore size under 2 µm, whereas the *p‐*PMMA shows a wide range of pore size (from ≈200 nm to 8 µm) (Figure 2F,G). To investigate the influence of pore structure on optical reflectance, electric field simulations on computational models of *p‐*SEBS and *p‐*PMMA are conducted using various wavelengths from 0.3 to 2.5 µm, with a step of 0.2 µm (Figures S4 and S5, Supporting Information). The simulation method is depicted in the Experimental Section.==

As representative results for solar spectrum and thermal wave regions, the electric fields corresponding to 0.5, 2.1, and 9 µm wavelength are shown in Figure 2H. At short wavelength (i.e., *λ* = 0.5 µm), the electric field propagation in *p‐*SEBS is deeper than that in *p‐*PMMA and thus *p‐*PMMA is more efficient in blocking the incident photons with 0.5 µm wavelength (Figure 2H; top). In contrast, the *p‐*SEBS shows superior light blocking compared to that of *p‐*PMMA at the longer wavelength (i.e., *λ* = 2.1 µm) (Figure 2H; middle). The *p‐*PMMA and *p‐*SEBS are useful to reflect light with visible and near‐infrared (NIR) wavelengths, respectively, because *p‐*PMMA mainly has abundant nanopores and *p‐*SEBS primarily includes microscale pores. These results have the same tendency with the previous report studying the scattering efficiencies of nano‐ and microscaled pores.^[^ ^39^ ^]^ In addition to improving the solar rejection, the coating of *p*‐PMMA on *p*‐SEBS is useful to reduce the surface reflection of thermal wavelength (i.e., *λ* = 9 µm) compared to *p*‐SEBS (Figure 2H; bottom). The electric field in *p*‐PMMA is stronger than that of *p*‐SEBS, which indicates low surface reflection of *p*‐PMMA. This can be explained by the antireflection effect resulting from the gradual refractive index of the abundant nanopores in *p*‐PMMA. Figure 2I experimentally demonstrates the exceptional antireflection effect of the *p*‐PMMA layer in the LWIR region. The reflectivity spectrum of NMVP with the light incident on the surface of *p*‐PMMA clearly exhibits almost zero reflection, indicating that NMVP with the top surface of *p*‐PMMA intensely radiates thermal wave compared to the surface of *p*‐SEBS.

Figure 2J spectrally demonstrates the following optical features: 1) *p‐*SEBS (optimum thickness ≈100 µm) has lower and higher reflectance in the visible and NIR ranges, respectively, and 2) *p‐*PMMA (optimum thickness ≈400 µm) has higher and lower reflectance at visible and NIR ranges, respectively. The detailed structural optimizations of *p‐*PMMA and *p‐*SEBS are shown in Figures S6 and S7 in the Supporting Information, respectively. Thicker *p‐*SEBS does not provide better optical reflectance owing to pore saturation (Figure S8A, Supporting Information). Although thicker *p‐*PMMA enhances optical reflectance, thicker one loses its flexibility because of the inherent rigidity of PMMA (Figure S8B–D, Supporting Information). See Videos S4 and S5 in the Supporting Information for full bending test process. In short, the NMVP (≈500 µm thickness) consisting of two porous polymers presents significantly improved reflectance spectra.

As shown in Figure 2H,I, a high surface reflection of *p*‐SEBS lowers the thermal emissivity (Figure 2K; red). Although *p*‐PMMA has antisurface reflection, the emissivity is still lacking for powerful thermal radiation (Figure 2K; green). However, the NMVP exhibits a near‐unity emissivity in the LWIR window (Figure 2K; blue). Thus, the additional *p‐*PMMA coating on *p‐*SEBS enhances not only solar reflection but also LWIR emission. Depending on humidity, the transmissivity in the LWIR window can vary remarkably (Figure S9A, Supporting Information). Based on its outstanding solar reflectivity and LWIR emissivity, the NMVP is expected to achieve exceptional cooling performance metrics such as cooling power and temperature with respect to humid and dry conditions (Figure S9B, Supporting Information). To demonstrate sub‐ambient cooling by the fabricated porous polymers, measurement setup was established (Figure S9C, Supporting Information). Thermocouples were attached to the backside of Cu objects to measure the temperature, and the porous polymers (i.e., *p*‐SEBS and NMVP) are laminated on the Cu films using an acrylate‐based adhesive that has high emissivity in the LWIR region. To minimize nonradiative heat‐exchange processes, a convection shield composed of low‐density polyethylene film covers on the setup. The detailed measurement setup and approach are presented in the Experimental Section.

The daytime measurement results confirm that NMVP achieves a lower temperature than *p‐*SEBS, although both samples obtain sub‐ambient cooling (Figure 2L). Temperatures of samples dropped around 12:00 in June 08, 2020 by sunlight blocking of clouds. Because the BE strongly absorbs the sunlight, the temperature drop of BE was the largest among all samples. The adhesive layer beneath *p‐*SEBS assists the thermal emission and hence it reduces the cooling performance gap between NMVP and *p‐*SEBS, as illustrated in Figure S9B in the Supporting Information. However, 5 d continuous temperature logging reliably demonstrates the superior cooling capability of NMVP compared to *p‐*SEBS with an adhesive layer (Figure S10, Supporting Information). The results are summarized in Figure 2M. NMVP is superior in *p‐*SEBS in reducing the object temperature under any weather conditions including hazy, humid, overcast, and clear days. In addition, NMVP exhibits a remarkable cooling performance in the tilted state, which should be considered for the integration in wearable devices. See Figure S11 in the Supporting Information for a detailed discussion on the cooling performance of slanted NMVP. Note S2 in the Supporting Information describes the detailed comparison result between WE based on commercial white dye and the optimized NMVP.

**Figure 3:**
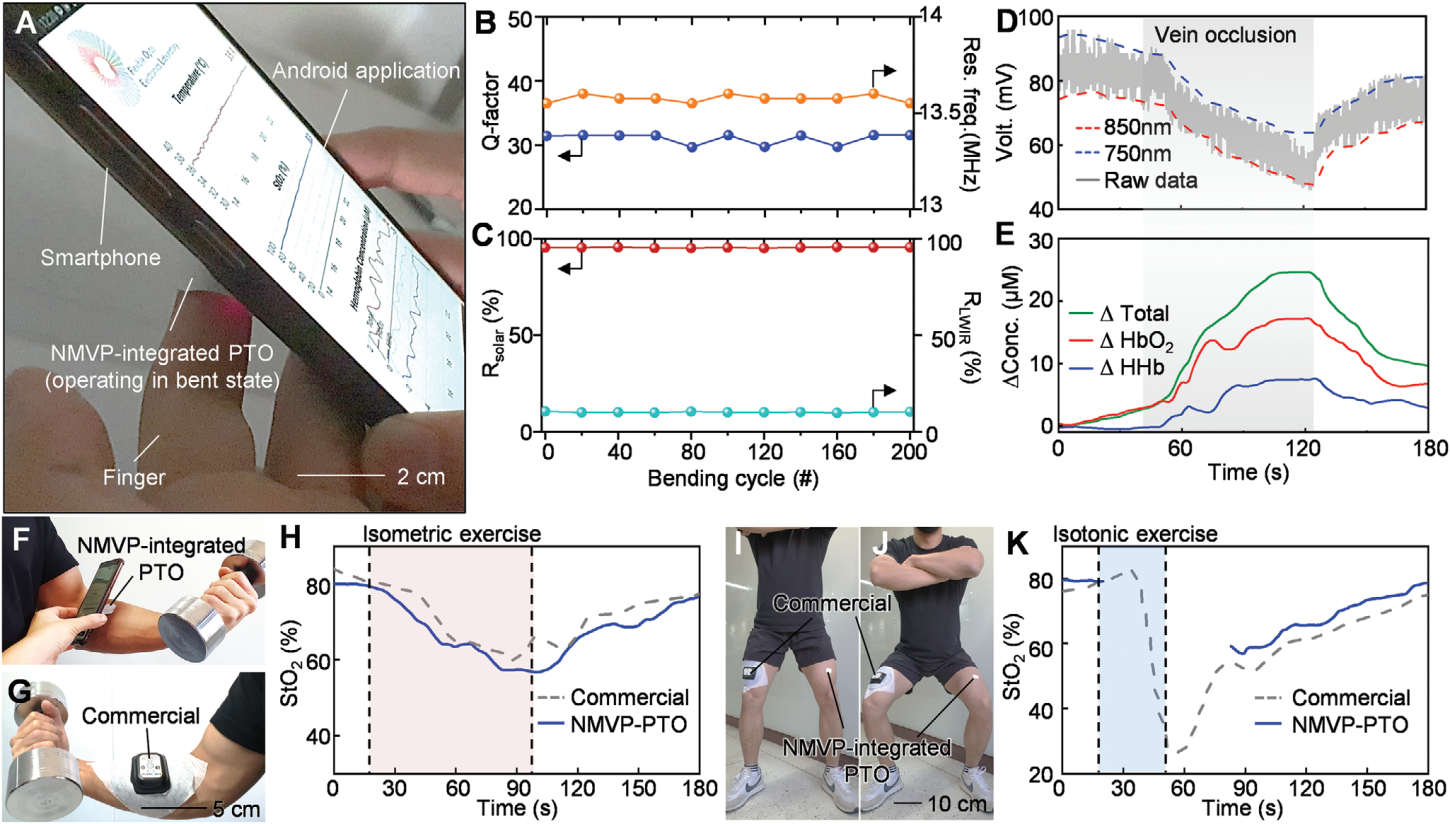
Device performance evaluation for flexibility and reliability. A) Photograph of NMVP‐integrated PTO on finger connected with a smartphone. B,C) Cyclic bending test result for 200 bending cycles in 20 increments for PTO and NMVP. B) Resonance frequency and Q‐factor of PTO with bending cycle. C) Average solar reflectance and LWIR emissivity of NMVP with bending cycle. D,E) Result of vein occlusion test with NMVP integrated PTO at forearm. D) Measured raw data and E) variation in hemoglobin concentration calculated by data processing. Photograph of subjects for isometric exercising at each side of forearm using dumbbell with F) NMVP‐integrated PTO and G) commercial device. H) StO_2_ measured by NMVP‐integrated PTO (blue line) and commercial device (gray dash line) during isometric exercise. I,J) Isotonic exercise with attached NMVP‐integrated PTO and the commercial device to each thigh. K) StO_2_ measured by NMVP‐integrated PTO (blue line) and commercial device (gray dash line) during isotonic exercise.

**Figure** **3**A shows the measurement of tissue oxygen saturation (StO_2_) and temperature using a smartphone with conformal contact of the NMVP‐integrated PTO to the finger (RoC 1.3‒1.8 cm).^[^ ^45^ ^]^ Detailed measurement results are displayed in Figure S12 in the Supporting Information. In addition, in the bent geometry, the frequency characteristics do not change significantly and the PTO can transfer the collected data to the NFC reader smoothly (Figure S12 and Video S1, Supporting Information). As shown in Figure 3B,C, cyclic bending tests of PTO and NMVP were performed, respectively. In the cyclic test, for 200 cycles, the measurements were repeated every 20 bends in terms of frequency features (i.e., Q‐factor and resonant frequency; Res. Freq.) and photonic features (i.e., average solar reflectance; *R*_solar_ and LWIR emissivity; *ε*_LWIR_). See Figure S13 in the Supporting Information for the detailed flexibility test of the NMVP. Therefore, we confirmed that, even in a bent state or after cyclic bending stress, there is no performance degradation of NMVP‐integrated PTO.

The conventional tissue oximeter allows the measurement of local tissue oxygenation, which serves to monitor peripheral blood circulation. The vein occlusion test was performed as an in vivo evaluation of PTO performance (Figure 3D,E).^[^ ^5^, ^46^ ^]^ During the venous occlusion of ≈70 s, the raw data measured by NMVP‐integrated PTO as well as the calculated variations in concentration of deoxyhemoglobin (ΔHHb), oxyhemoglobin (ΔHbO_2_), and total hemoglobin (ΔTotal) showed similar trends as the reported data,^[^ ^5^, ^46^ ^]^ which are based on the principle of blood accumulation during venous occlusion. The measurement method is described in the Experimental Section.

**Figure 4:**
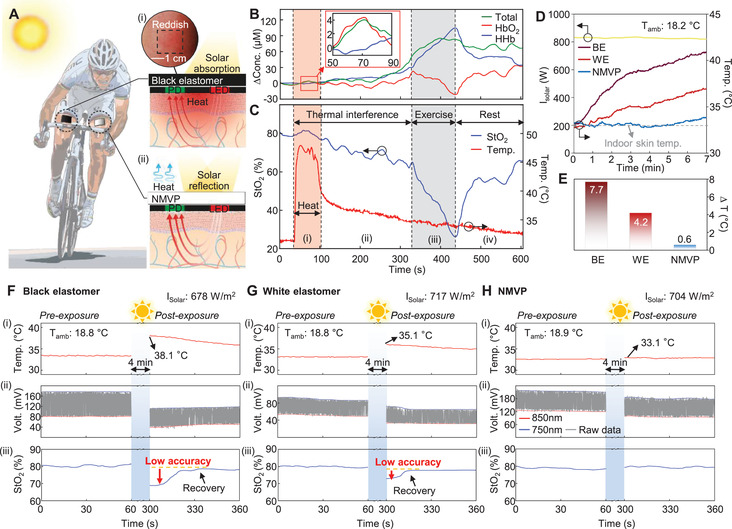
Effect of heat on tissue oximeter and thermal protection by NMVP. A) Illustration image of cyclist with NMVP‐integrated and black elastomer‐integrated tissue oximeters on each thigh. The insets represent thermal effects: i) light absorption of black elastomer. The red gradation presents the heat transfer from black elastomer to device and skin. The inset image shows black‐elastomer‐covered skin with postexposure to direct sunlight, which turned reddish. ii) Thermal effect‐free NMVP. The sky‐blue arrows represent thermal radiation from NMVP. Investigation of the thermal interference with measuring B) hemoglobin concentration and C) StO_2_. The red and gray areas indicate heating and exercising periods, respectively. D) Measured temperatures of NMVP, BE, and WE on skin under direct sunlight for 7 min. The dashed line expresses the indoor measured result (≈32.8 °C). E) The difference between indoor and outdoor skin temperatures. The measurement was performed on November 15, 2020. Comparison of measurements pre‐exposure and postexposure with F) BE‐integrated device, G) WE‐integrated device, and H) NMVP‐integrated device attached to forearm. Sky‐blue regions represent time period (4 min) of direct sunlight exposure. The measurements were performed on November 15, 2020.

As shown in Figure 3F–K, exercise experiments on subjects were performed to compare NMVP‐integrated PTO with a commercial tissue oximeter. Reliability of commercial device was demonstrated by comparison with the golden standard measurement (VO_2_ max).^[^ ^47^ ^]^ Subjects performed isometric and isotonic exercises to impose muscle fatigue at the forearm and thigh, respectively. Commercial sensors and NMVP‐integrated PTO were attached to each side of the forearm and thigh. During ≈100 s of isometric contraction at each forearm (highlighted in red), as the variation in oxygen consumption of muscle,^[^ ^47^, ^48^ ^]^ the StO_2_ measured by NMVP‐integrated PTO decreased to ≈60% and recovered to normal levels after the end of the exercise (≈80%), showing similar trends to the commercial device (Figure 3F–H). In addition, after ≈30 s of isotonic exercise for the thigh (highlighted in blue), O_2_ consumption gradually normalizes; as a result, StO_2_ recovers to the normal level (≈80%) with similar trends to the commercial device (Figure 3I–K). Therefore, we demonstrate the reliability of NMVP‐integrated PTO by comparing it with a reliable commercial device. The details of the exercise experiments can be confirmed in the Experimental Section and Figure S14 in the Supporting Information. **Figure** **4**A is a conceptual illustration of a cyclist, with NMVP‐ and black elastomer‐integrated PTO attached to each thigh to evaluate muscular endurance in daytime. Because the black elastomer applied to block ambient light absorbs solar energy, it results in a thermal effect in the body (i.e., blood flow variation^[^ ^18^, ^19^ ^]^ and tissue deoxygenation^[^ ^20^, ^21^, ^22^ ^]^) as well as optoelectronic device degradation^[^ ^23^, ^24^, ^25^, ^26^ ^]^ (Figure 4Ai). On the contrary, as NMVP can effectively reflect sunlight and radiate thermal energy, it not only blocks ambient light but also provides thermal protection of the device (Figure 4Aii).

To investigate the thermal interference with the tissue oxygenation measurement, a heating experiment is conducted with a hairdryer to confirm the local heating effect at the measurement site on the biosignal of the tissue oximeter (Figure 4B,C). While heating to a maximum temperature of ≈45 °C, the ΔHbO_2_ and StO_2_ temporarily increase, because blood flow increases with vasodilation.^[^ ^18^, ^19^ ^]^ Following the heating, ΔHHb is gradually elevated, and accordingly StO_2_ is moderately decreased, due to increased O_2_ consumption^[^ ^20^, ^21^, ^22^ ^]^ and deterioration of LED performance^[^ ^23^, ^24^, ^25^, ^26^ ^]^ originated from intense heating. In this state, the subject performed the exercise, and the difference between ΔHbO_2_ and ΔHHb as well as StO_2_ change dramatically, owing to the synergy of exercise and thermal interference. During the rest period, although StO_2_ recovered, it requires a sufficient time to recover to the normal level. The undesired heat gain causes the phenomena such as blood flow variation,^[^ ^18^, ^19^ ^]^ tissue deoxygenation,^[^ ^20^, ^21^, ^22^ ^]^ and optoelectronic device degradation,^[^ ^23^, ^24^, ^25^, ^26^ ^]^ hence such complicated event is referred to the thermal interference leading to inaccurate measurement.

As displayed in Figure 4D,E, after 7 min of exposure to direct sunlight, the temperatures of skin covered by the BE and WE increase to ≈41 and ≈37 °C, respectively; in contrast, the temperature of the area covered by the NMVP remains at ≈33.4 °C, which is similar to the temperature indoors (≈32.8 °C). Figure 4F–H compares the StO_2_ measurements of BE‐integrated, WE‐integrated, and NMVP‐integrated PTO in the pre‐exposure and postexposure states for 4 min under direct sunlight on the same day. The temperature of the BE‐integrated PTO rose to 38.1 °C; accordingly, the raw data changed significantly and the StO_2_ decreased from the normal level to ≈69% (Figure 4F). As displayed in Figure 4G, although the temperature of the WE‐integrated PTO increased to 35.1 °C lower than in the first case, the thermal interference affects the raw data and the StO_2_ result (≈73%). In contrast, the temperature of the NMVP‐integrated PTO barely increased even under sunlight exposure, blocking the thermal interference perfectly; correspondingly, the StO_2_ maintained a normal level (Figure 4H). Additionally, as a result of the comparison experiments implemented in summer, severe thermal interference was observed (Figure S15, Supporting Information). These results demonstrate that the tissue oximeter should be protected from outdoor heat sources to provide an accurate StO_2_ measurement; otherwise, it cannot be used for outdoor applications. The proposed NMVP‐integrated PTO ensures reliable StO_2_ readings in outdoor environments as it remains in thermal protection.

In this study, we have presented a new class of tissue oximeter, free of thermal concerns, by integrating NMVP and a self‐produced patch‐type tissue oximeter. Our PTO has the advantages of wireless operation and flexible form factor for user comfort. Optically optimized NMVP of thickness 500 µm, which is fabricated using a self‐assembly method, contacts with the skin conformably. Additionally, we spectrally characterized the fabricated NMVP, and theoretically evaluated its cooling performance. Temperature measurements under various weather conditions confirmed the outstanding cooling performance of NMVP. In a comparison experiment with the commercial device, we verified that the PTO can provide reliable measurement in tissue oxygenation. Additionally, we demonstrated and analyzed undesired changes in the StO_2_ results that occurred from local heating. A comparison of the NMVP‐, white polydimethylsiloxane (PDMS)‐, and black elastomer‐integrated PTO showed that the NMVP‐integrated PTO provided accurate results, eliminating the thermal interference outdoor. Therefore, by integrating NMVP with the PTO, we developed an outdoor‐usable sports assistant patch‐type device, which mitigates the effects of undesired internal and external heating sources such as the device itself and sunlight.

## 3 Experimental Section

The experimental details, including structural optimization, device operating principle and fabrication, NMVP fabrication, device radio frequency (RF) property analyses, bending test of PTO and NMVP, data acquisition method, optical simulation, in vivo experiment (vein occlusion and exercise experiments), and indoor/outdoor measurements, are provided in the Supporting Information.

## Conflict of Interest

The authors declare no conflict of interest.

## Supporting information

Supporting Information

Supplemental Video 1

Supplemental Video 2

Supplemental Video 3

Supplemental Video 4

Supplemental Video 5

Supplemental Video 6

## Data Availability

Research data are not shared.
