# Supplementary material for: Outdoor‐Useable, Wireless/Battery‐Free Patch‐Type Tissue Oximeter with Radiative Cooling
Source: Adv Sci (Weinh). 2021 Mar 9;8(10):2004885. doi: 10.1002/advs.202004885 (PMC8132059; doi:10.1002/advs.202004885)
Supplement: Supplementary file 1 — Supporting Information [file ADVS-8-2004885-s005.pdf]

© 2021 Wiley-VCH GmbH

ADVANCED SCIENCE
Open Access

Supporting Information

for *Adv. Sci.*, DOI: 10.1002/advs.202004885

Outdoor-useable, Wireless/Battery-free Patch-type Tissue Oximeter with Radiative Cooling

Min Hyung Kang[1], Gil Ju Lee[1], Joong Hoon Lee[1], Min Seok Kim[1], Zheng Yan[2,3], Jae-Woong Jeong[4], and Young Min Song[1,5,6]*

Supporting Information

Outdoor-useable, Wireless Patch-type Tissue Oximeter with Radiative Cooling

Min Hyung Kang†, Gil Ju Lee†, Joong Hoon Lee, Min Seok Kim, Zheng Yan, Jae-Woong Jeong, and Young Min Song*

Experimental Section

Device Fabrication: As can be seen in **Figure S16**, an NFC coil and circuit was fabricated by photolithography and wet etching to pattern both sides of a Cu (18 μm)/polyimide (25 μm)/Cu foil (18 μm) flexible printed circuit board (FPCB, SME, South Korea). The electrical components (i.e., NFC chip, μ-controller, light-emitting diode (LED), photodiode (PD), thermistor, resistor, and capacitor) are soldered with silver paste on the circuit. The via-holes for connecting the top and bottom coil are formed by drilling holes with micro drill bit, and soldering. Black encapsulation layer is silicone elastomer (Q1-4010, Dow Corning, USA) with adding ~10 wt% black dye (Silcpig, Hyupshin, South Korea). Biocompatible adhesive (PC2723U, ScapaHealthcare, UK) was applied to the side of the device in contact with skin.

Components: The main components are as follows: NFC chip (RF430FRL152H, Texas Instruments, USA, 4.1×4.1×1 mm^3), μ-controller (ATtiny10, Atmel, USA, 2×2×0.6 mm^3), 750 nm LED (L1CU-FRD1, Lumileds, The Netherlands, 2×2×1 mm^3), 850 nm LED (15406085BA300, Wurth Elektronik, Germany, 1.6×0.8×0.6 mm^3), PD (VEMD1060X01, Vishay, USA, 2×1.25×0.85 mm^3), and thermistor (NCU18WF104D6SRB, Murata Electronics, Japan, 1.6×0.8×0.8 mm^3).

RF Analysis of Device: The resonance frequency (f_{res}) of the coil on the device was measured using a vector network analyzer (VNA) (E5071C ENA, Keysight, USA) with a RF near-field probe (PBS1, AARONIA, Germany). The S11 magnitude mode was set to measure the f_{res} and Q-factor of the coil. The f_{res} of the coil is the frequency resulting in the minimal reflected power, as shown in **Figure S4**. The f_{res} equation and Q-factor equation were used to design the coil, which can be expressed as

$$f_{res} = \frac{1}{2\pi\sqrt{L_c C_c}} \qquad \textbf{(S1)}$$

$$\text{Q-factor} = \frac{1}{R_c}\sqrt{\frac{L_c}{C_c}} \approx \frac{f_{res}}{f_2 - f_1} \qquad \textbf{(S2)}$$

where L_c is the inductance of the coil and C_c is the total capacitance of the device. The Q-factor is determined by L_c, C_c, and the coil resistance (R_c). The Q-factor represents the sharpness of the resonance peak, and thus can be defined as an approximate calculation, where f_2 and f_1 is −3 dB frequency of f_{res} and $f_2 - f_1$ is the bandwidth of f_{res}.

Fabrication of NMVP: The optimized NMVP is prepared by a two-step phase-inversion process: 1) Drop-casting porous styrene-ethylene-butylene-styrene (*p*-SEBS) layer on slide glass and 2) spray-coating porous polymethylmetacrylate (*p*-PMMA) layer on the *p*-SEBS layer (**Figure S17**). For *p*-SEBS, 55.2 g of chloroform (C2432, Sigma-Aldrich) is mixed with 10.6 g of IPA and 2.4 g of SEBS beads (Tuftec™ H1062, Asahi Kasei, Japan) into a test tube at room temperature. For *p*-PMMA, 50 g of acetone is mixed with 5 g of DI water and 4 g of PMMA beads (182230-500G, Sigma-Aldrich). The solutions are then sonicated for one day to completely dissolve the SEBS and PMMA beads.

After the complete dissolution, the SEBS solution is dropped on a glass slide, and then the solvent (*i.e.,* chloroform) and nonsolvent (*i.e.,* IPA) are allowed to evaporate; thus,

this procedure generates the *p*-SEBS layer by forming a polymer chain containing air voids. An air-brushing tool is used to spray the *p*-PMMA solution on the formed *p*-SEBS layer. The spraying is performed from a distance of ~10 cm for 10 s. During and after the spraying step, similar to the *p*-SEBS layer, the solvent (*i.e.,* acetone) and nonsolvent (*i.e.,* DI water) are evaporated, and the PMMA produces porous polymer chains.

Bending test of PTO and NMVP: A cyclic bending test was performed on PTO and NMVP with cylindrical objects of ~1.3 cm RoC for 200 bending cycles in 20 increments, respectively. The frequency characteristics of PTO were measured every 20 bending repetitions with VNA. The solar reflectance and LWIR emissivity of NMVP were also measured every 20 bending cycles. Additionally, in order to evaluate the performance of the PTO in the bent state, the frequency characteristic was measured in the bent state, and the StO_2 and temperature measurements were compared before and after the bending of PTO on the finger (**Figure S12**). After measuring StO_2 and temperature for a certain period of time by placing a flat PTO on a finger, the PTO was bent on the finger for measuring StO_2 and temperature in a bent state.

Raw Data Processing for Tissue Oxygenation: The tissue oximeter data analysis was performed using commercial software (MATLAB R2013b, MathWorks Inc, USA). The smartphone Android application was designed using commercial software (Android Studio, Google, USA) based on JAVA program language. The raw data detected by the thermistor and PD are collected at a sampling rate of ~12.5 Hz. The two LEDs blinked with a frequency of 12 Hz. A local minima–maxima finding algorithm was used to separate the two levels of the LED signals. The extracted signals were reassembled by spline interpolation of each LED signal. In order to remove high-frequency noise, a low-pass filter (0.1 Hz, 10th-order Butterworth digital filter). The filtered data were calculated as optical densities as follows:

$$\mathrm{OD}\ (\lambda, t) = -\ln\left(\frac{I_t(\lambda)}{I_0(\lambda)}\right) \tag{S3}$$

where $I_t(\lambda)$ is the time-dependent signal and $I_0(\lambda)$ is the initial value of $I_t(\lambda)$, which were calculated for both wavelengths (λ_1 (850 nm) and λ_2 (750 nm)). In order to obtain the ΔHbO_2 and ΔHHb, the modified Beer-Lambert law for diffusive media was applied.[1]

$$\begin{bmatrix} \Delta HbO_2 \\ \Delta HHb \end{bmatrix} = \frac{1}{\rho} \begin{bmatrix} \varepsilon_{HbO_2}(\lambda_1)\cdot DPF(\lambda_1) & \varepsilon_{Hb}(\lambda_1)\cdot DPF(\lambda_1) \\ \varepsilon_{HbO_2}(\lambda_2)\cdot DPF(\lambda_2) & \varepsilon_{Hb}(\lambda_2)\cdot DPF(\lambda_2) \end{bmatrix}^{-1} \cdot \begin{bmatrix} OD(\lambda_1) \\ OD(\lambda_2) \end{bmatrix} \tag{S4}$$

where ρ is the interoptode distance (9 mm) and DPF(λ) is the differential path length factor depending on the wavelength.[2] The ε(λ) are extinction coefficients of HbO_2 and Hb.[3] StO_2 was calculated as follows:

$$StO_2 = \frac{HbO_2(t)}{HbO_2(t) + HHb(t)} = \frac{\left(Hb_t\,(t{=}0)\cdot StO_2(t{=}0) + \Delta HbO_2(t)\right)}{\left(Hb_t(t{=}0)\cdot StO_2(t{=}0) + \Delta HbO_2(t)\right) + Hb_t(t{=}0)\cdot\left(1 - StO_2(t{=}0)\right) + \Delta HHb(t))} \tag{S5}$$

The initial values of the total hemoglobin concentration at the forearm are (Hb_t) (0.08 mM) and StO_2 (at t = 0, 80%), respectively.[4, 5] Because the calculation method is driven by the change in oxygen saturation of tissues based on the change in optical density compared to the initial value, it cannot be used as a gold standard for measuring the exact tissue oxygen saturation of a subject. However, it can be used for healthcare purposes by observing the change in tissue oxygenation in a local area as changes occur in the body (e.g., exercise or temperature). For reliable data processing and plotting of StO_2, raw data must be accumulated for at least 3 s. In the Android application, by processing accumulated real-time data, StO_2 can be plotted at least every 3 s, and the data accumulation time can be adjusted.

Optical Simulation: Commercial software (FullWAVE, RSoft Design Group,

Synopsys, USA) based on a two-dimensional finite-domain time-difference (FDTD) algorithm is utilized to simulate the electric field distributions of *p*-SEBS and *p*-PMMA for different wavelengths. The pore analysis is conducted using the software package ImageJ (NIH, USA). Based on the average pore size and variance from scanning electron microscopy (SEM) images, random air void structures are generated with the dimensions of 50 × 10 μm^2 in the x and z directions for *p*-SEBS and *p*-PMMA, respectively, using MATLAB R2018A (MathWorks, Inc., USA). The generated random air voids are inserted as the refractive index profiles in the wave analysis tool. The boundary condition is defined as a perfectly matched layer (PML) to absorb all the outgoing radiation. A plane wave with wavelengths ranging from 300 to 2500 nm for solar spectrum and 9 μm for LWIR range is launched from a distance of 5 μm from the surface of the generated random air void structures. The refractive indices of polymer backgrounds for *p*-SEBS and *p*-PMMA are set to a constant value of 1.5. The air voids were assigned a refractive index of 1.0. In the simulation, a square grid size of 10 nm was used to calculate numerically stable results.

Spectral and Structural Characterization: The reflectance of the fabricated samples is characterized with an integrating sphere over the wavelength range of 280–2500 nm using an ultraviolet-visible-near-infrared spectrophotometer (Lambda 950, Perkin Elmer, Inc., USA). A Fourier transform infrared spectrometer (VERTEX 70v, Bruker, USA) with an Au-coated integrating sphere is used to obtain the emission property. The emissivity spectra are evaluated from 100 – R + T, which are the measured reflectivity and transmittance spectra. SEM (S-4700, Hitachi Hi-Tech, Japan) is exploited to observe the perforated structures in polymers.

Energy Balance Equation: Using the measured emissivity spectra, the cooling temperature and cooling power are estimated by MATLAB R2018A (MathWorks, Inc., USA)

based on the thermal equilibrium equation given as follows:

$$P_{rad}\left(T_{Sample}\right)\text{-}P_{Sun}\text{-}P_{atm}\left(T_{ambient}\right)+h_c\left(T_{sample}\text{-}T_{ambient}\right) \quad \textbf{(S6)}$$

where $P_{rad}(T_{sample})$ is the power radiated by the structure per unit area, $P_{atm}(T_{ambient})$ is the absorbed power per unit area from the atmosphere, P_{Sun} is the incoming solar power absorbed by the structure per unit area, and $h_c(T_{sample}–T_{ambient})$ is the conductive and convective heat exchange powers. These four terms are evaluated using

$$P_{rad}\left(T_{Sample}\right)=\int_0^{2\pi}\int_0^{\pi/2}\int_0^{\infty} I_{BB}\left(T_{sample},\lambda\right)\epsilon(\lambda,\theta)\cos(\theta)\sin(\theta)\, d\lambda d\theta d\phi \quad \textbf{(S7)}$$

$$P_{atm}\left(T_{ambient}\right) = \int_0^{2\pi}\int_0^{\pi/2}\int_0^{\infty} I_{BB}\left(T_{ambient},\lambda\right)\epsilon(\lambda,\theta)\epsilon_{amb}(\lambda,\theta)\cos(\theta)\sin(\theta)\, d\lambda d\theta d\phi \quad \textbf{(S8)}$$

$$P_{Sun}=\int_0^{\infty} I_{AM1.5G}(\lambda)\epsilon(\lambda,\theta)d\lambda \quad \textbf{(S9)}$$

Here, $I_{BB}=(2hc^2/\lambda^5)/[e^{\frac{hc}{\lambda k_B T}}\text{-}1]$ is the spectral radiance of a blackbody at temperature T, where h, c, k_B, λ, and h_c are Planck's constant, the velocity of light, the Boltzmann constant, the wavelength, and the nonradiative heat-exchange coefficient, respectively. The atmospheric emissivity is given by $\varepsilon_{amb}(\lambda,\theta)=1\text{-}t(\lambda)^{1/\cos(\theta)}$, where t is the sky transmission calculated by MODTRAN 6 using the conditions of an urban site at mid-latitude in summer (*i.e.,* humid LWIR window). To calculate P_{Sun}, the solar irradiation is expressed by the AM1.5G Global Tilt spectrum with an intensity of 1000 W/m^2.

In Vivo Experiment: Before proceeding with the experiment, the subject was allowed to acclimate to the environment for approximately 120 s, sitting comfortably. The device was attached to the forearm and thigh, and the bio-signals were captured by an NFC reader at a distance of ~ 0.7 cm. The maximum communication distance between the NFC reader and PTO is ~ 1 cm (**Video S6**). All of the in vivo experiments in this study were performed in

compliance with the protocol approved by the institutional review board at Gwangju Institute of Science and Technology (GIST). Four healthy subjects, aged 25-30 years, participated in the study. Informed consent was obtained from all subjects involved in the study.

Vein Occlusion Test: A standard method for assessing peripheral vascular diseases is venous occlusion test. An inflating cuff on the subject's bicep temporarily occluded venous blood flow for ~80 s, the variation in the measured signal at the forearm was monitored by our tissue oximeter integrated with NMVP.

Exercise experiment with commercial device: As shown in **Figure 3F–3K** and **Figure S14**, The NMVP-integrated PTO was compared with a commercial device (MOXY, Fortiori Design, USA). The NMVP-integrated PTO and commercial device were attached to each forearm and thigh. For isometric exercise, the subject held ~5 kg of dumbbell on each arm and endured for ~100 s. For isotonic exercise, the subject performed 15 squats, after which, StO_2 of the thigh with NMVP-integrated PTO was measured. The commercial device continuously measured the StO_2 of the opposite thigh.

Convection heating experiments: The experiment was executed for 605 s, corresponding to (i) 60 s heating (highlighted in red) with (ii) 220 s rest and (iii) 100 s exercise (highlighted in gray) with (iv) 160 s rest (**Figure 4B** and **4C**) at forearm. A hairdryer was used for heating, which heats around the measurement site attached to the device. The exercise is an isometric contraction at the forearm with a ~ 5 kg of dumbbell.

Indoor and Outdoor Tissue Oxygenation Comparison: The StO_2 was measured indoors for ~60 s, followed by outdoor exposure for ~4 min under direct sunlight, after which the StO_2 was measured again for ~60 s for comparison. In order to compare the StO_2 changes indoors and outdoors, the I_0 (λ) value measured pre-exposure was used to calculate the outdoor StO_2 value. The experiment was performed on November 14, 2020.

Outdoor Measurement of Cooling Temperature: For the radiative cooling temperature measurement, the back surfaces of the samples are laminated with Cu foils of the same size as the samples. Between the samples and Cu foils, adhesive temperature sensors (ST-50, RKC Instrument Inc., Japan) are inserted and connected to a data logger (RDXL6SD, Omega Engineering, USA). An ambient air sensor was inserted in an Al-coated paper box with air flow to measure the temperature of the naturally convective air. This box prevents the self-heating of the ambient air sensor by solar irradiance. A pyranometer (CMP, Kipp & Zonen, The Netherlands) is placed next to the measurement setup to record direct and diffuse solar irradiance.

Supplementary Note 1. Ray-tracing simulation for PTO design

The distance between the LEDs and the PD, namely the interoptode distance, sets the penetrat ion depth of backscattered light into the skin. Generally, increasing the interoptode distance e xtends the light penetration depth[6] and enlarges the probing volume, thus enhancing the sens itivity.[1, 7] However, an increase in interoptode distance decreases the light intensity arriving at the PD owing to absorption and scattering of tissue, water, melanin, and fat, which increase s the signal-to-noise ratio of the detected signals.[7] Therefore, considering the light intensity and noise received from PD, we set the interoptode distance as 9 mm based on a commercial non-sequential ray-tracing simulation (OpticStudio 16.5, ZEMAX LLC., USA).

Figure S2A and S2B display the simulation domain of the designed structure in the top view and side view. The background material was set to 'skin' by using Henyey-Greenstein phase function with optical constants such as absorption coefficient, scattering coefficient, anisotropic factor, and refractive index (**Table S3**).[8, 9] To investigate the dependence of light power on the distance of the photodiode, we set seven rectangular detectors with distances of 3, 5, 7, 9, 11, 13, and 15 mm from the two LEDs. In addition, to mimic the light shielding of NMVP, we placed a diffuse reflector on the black encapsulation layer as the light shield layer. The black encapsulation layer was simplified by a perfect absorber.

$$\text{Noise ratio} = \frac{P_\mathrm{e}}{P_\mathrm{t}} \qquad \textbf{(S10)}$$

Noise ratio was defined as the ratio of light penetrated through only the epidermis without blood vessels (P_e) to light penetrated through the epidermis into the dermis with blood vessels (P_t). In order to calculate P_e, we considered the optical properties of a 0.1-mm-thick epidermis and reflection at the contact interfaces between the epidermis and dermis. In the case of P_t, the optical properties of both 0.1-mm-thick epidermis and 2-mm-thick dermis were considered. Based on the simulation, when the interoptode distance was more than 9 mm, the scattered light was quenched, and thus, the noise ratio was 0. Moreover, with increasing interoptode distance, the amount of light reaching the PD decreases (**Figure S2**C and **S2**D). As can be seen in the contour map of **Figure S2**E and **S2**F, the light scattered only into the epidermis is almost extinguished at a distance greater than the interoptode distance of 9 mm. Therefore, we designed an interoptode distance of 9 mm so that we not only achieved a lower noise ratio, but also allowed large amounts of light to reach the PD.

Supplementary Note 2. Comparison between white elastomers and NMVP

A commercial white dye based on TiO_2 nanoparticles is the easiest and most affordable material that can be used to fabricate white-colored radiators. In our study, we also compared the optical and cooling features between the white elastomer (WE) and NMVP. For this, we first fabricated WE with polydimethylsiloxane (PDMS) by doping different white dye densities (Silc Pig^{TM}; White, Smooth-on, USA). The doping densities were 0.2, 0.4, 0.6, and 0.8 mL with a fixed PDMS weight (11g; 10:1 ratio of base elastomer and curing agent). Based on these ratios, we made sufficiently thick white PDMSs (~ 3.6 mm) to confirm the optical and cooling features, excluding the thickness effect (**Figure S18A**). Figure S19A displays the thickness difference between the white PDMSs and NMVP. The NMVP has a thickness of ~ 0.2 mm.

The configuration of the individual temperature measurement unit is described in **Figure S18B**. A commercial blackbody absorbs sunlight through the samples. A temperature sensor measures the temperature of the blackbody, and the bottom Styrofoam thermally isolates the sample from the ground. **Figure S18C** shows the measured temperatures of the four white PDMSs and NMVP. The NMVP maintains the lowest temperature compared to other white PDMSs. Interestingly, all white PDMSs show a similar temperature regardless of the white dye density. Because the fabricated white PDMSs has a sufficiently thick layer, the density of the white dye does not significantly affect the optical feature. **Figure S18D** demonstrates the above-mentioned discussion using the reflectance spectra of white PDMSs and NMVP. The white PDMSs exhibit intense absorption at ultraviolet (UV) wavelengths under 400 nm. In the near-infrared region (NIR), the absorption of white PDMSs is strong. However, the NMVP shows more powerful reflection than white PDMSs in both spectral regions. Owing to its exceptional optical feature, NMVP sustains lower temperatures than all white PDMSs.

To investigate the influence of thickness on white PDMS, we also fabricated three white PDMSs with thicknesses of ~ 190, 440, and 630 μm. The PDMS weights and white dye amounts were fixed at 11 g (10:1 ratio of base elastomer and curing agent) and 0.8 mL, respectively. **Figure S19A** exhibits photographs of three white PDMSs and NMVP. Notably, thin white PDMSs such as ~ 190 and 440 μm cannot sufficiently block the visible light. The bottom blackbody is seen through the white PDMSs. However, the blackbody is not seen in thick white PDMS and NMVP. **Figure S19B** displays the measured reflectance spectra of three white PDMSs and NMVP in the solar spectrum range. Depending on the thickness, the reflectance of the white PDMS varies remarkably. In particular, the white PDMS with a thickness of ~ 630 μm, which has a three-fold thickness compared to that of NMVP, has poor reflectance compared to that of NMVP in all ranges. The measured temperature results show that white PDMSs with thicknesses of ~ 190, 440, and 630 μm cannot achieve daytime radiative cooling, whereas the NMVP shows daytime cooling under strong solar intensity, ~ 1000 W/m^2 (**Figure S19C**).

Based on the above experiments, the commercial white dye-doped elastomer cannot achieve daytime radiative cooling at a thickness of ~ < 630 μm. In addition, the sufficiently th ick white elastomers cannot obtain a notable radiative cooling performance than NMVP beca use of the strong absorptions in the UV and NIR regions. However, the blackbody NMVP sh ows daytime cooling under strong sunlight (~ 1000 W/m^2). Therefore, the NMVP is the best material that can be used to realize thermally protected wearable optoelectronics.

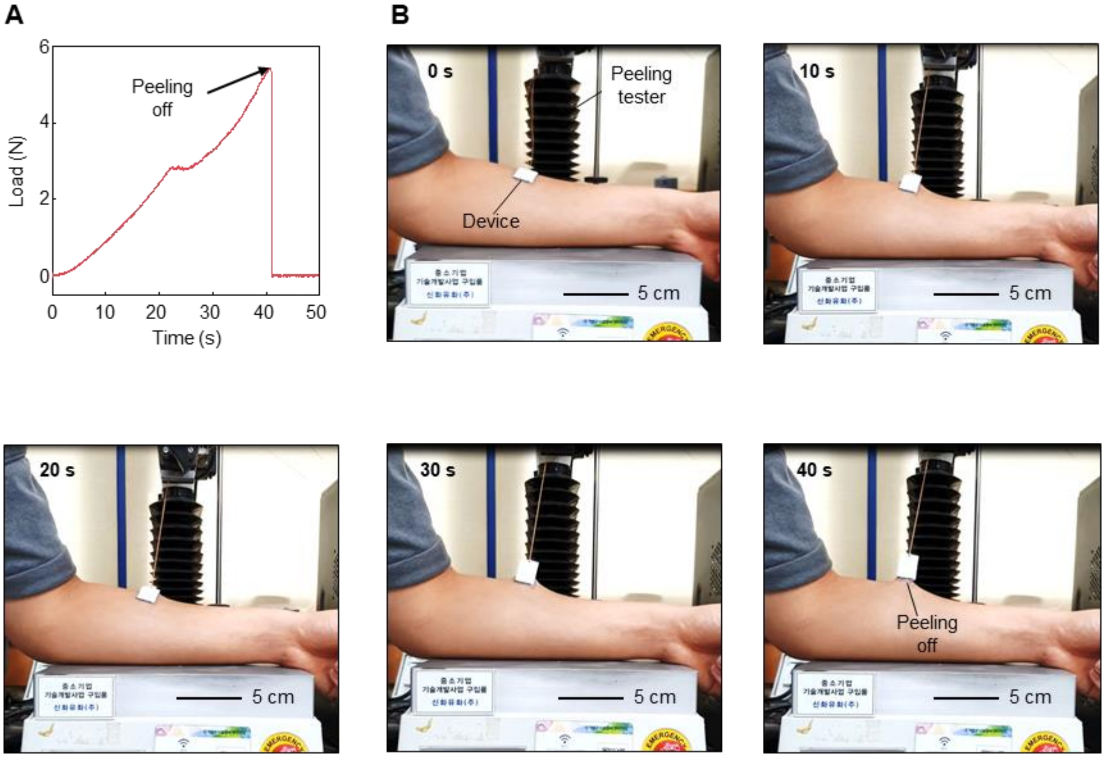

Figure S1. Result from mechanical measurement obtained using adhesion tester (text analyzer pulse, Micro stable, UK) on devices mounted on the forearm. (A) Result of adhesion test. (B) Photos were taken at 10 s intervals during the adhesion test. The device peeled off from skin on ~40 s.

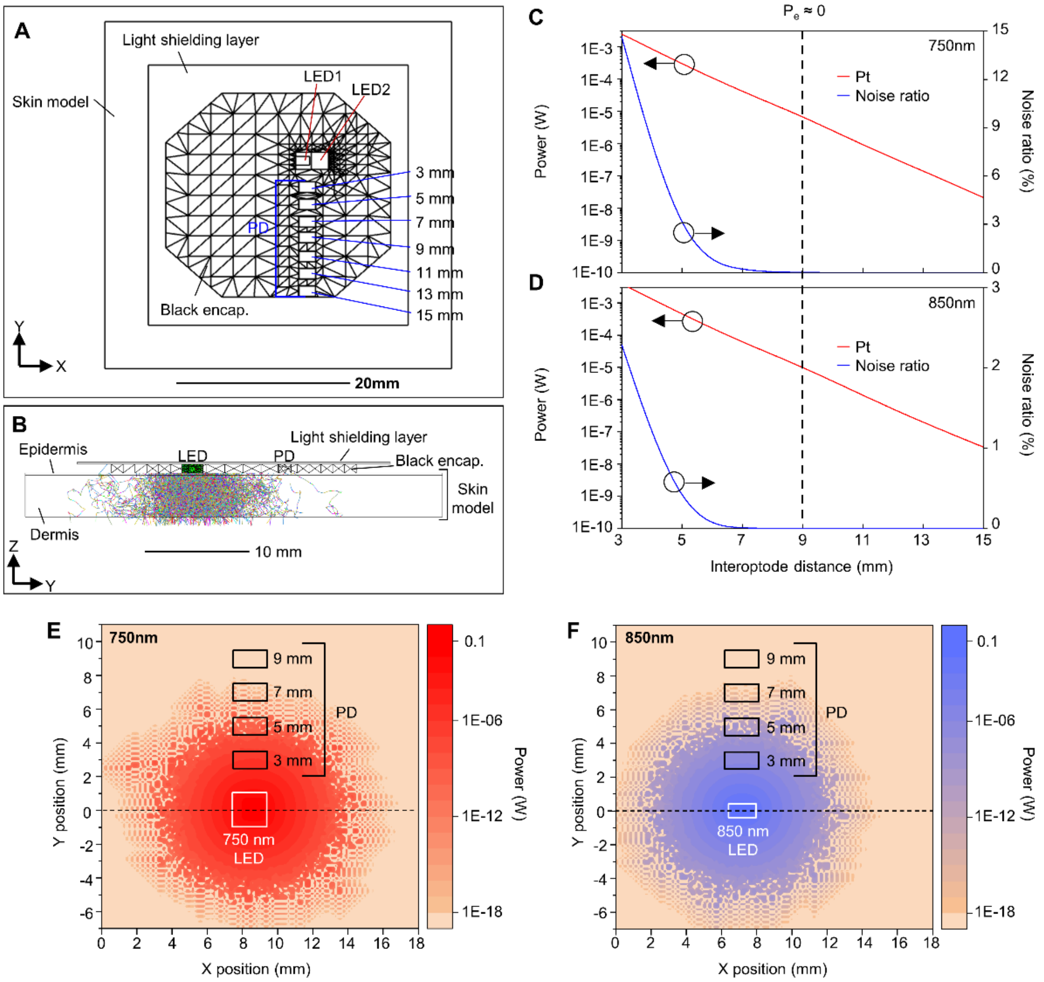

Figure S2. Ray-tracing simulation for interoptode distance. (A) Top view and (B) side view of simulation domain of the designed structure including skin model, NMVP, black encap, LEDs and PDs. (C, D) Noise ratio and light intensity detected by PD for light sources with the wavelength of (C) 750 nm and (D) 850 nm. (E, F) Contour maps showing scattered and spread light through the epidermis from the wavelengths of (E) 750 nm and (F) 850 nm.

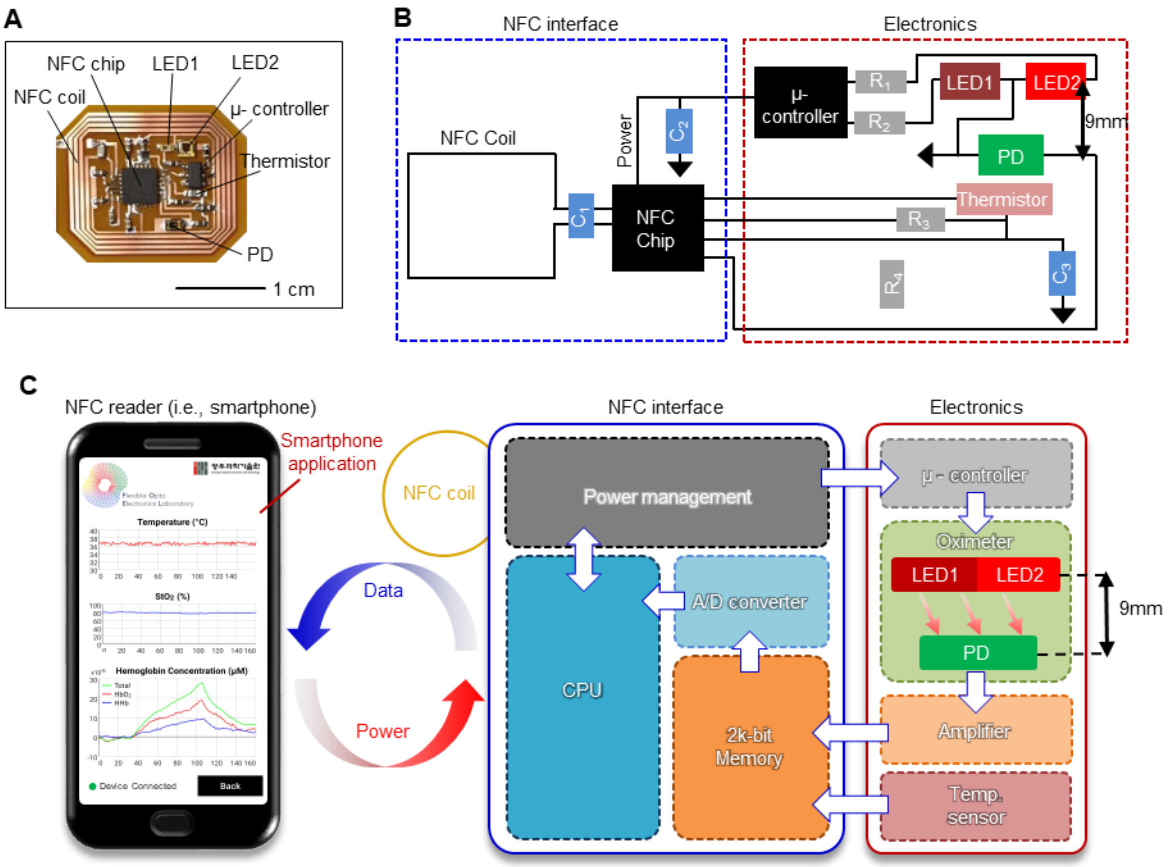

Figure S3. Wireless/ battery-free NFC system based tissue oximeter. (A) Photograph image of unecapsulated device. Wavelength of LED1 and LED2 is 850nm and 750nm, respectively. (B) Circuit diagram of the device. R_1 (1.1 kΩ) and R_2 (1.2 kΩ) are LED resistor. R_3 (100 kΩ) is reference resistor for thermistor. R_4 (5 MΩ) is amplification resistor. C_1 (9 pF) is resonance capacitor for resonance frequency tuning of NFC system. C_2 (0.1 μF) and C_3 (1 μF) are decoupling capacitor to remove noise. (C) Block diagram of device system to explain principle of device operation. The acquired data by the customized smartphone application is inserted in the left of Figure S3C. The device operating system is functionally classified into three parts: the first part is the NFC reader (i.e., smartphone) that transfers power to the device and collects data from the device. The second part is the NFC interface composed of an NFC module (i.e., NFC chip, RF coil). The third part is the detection part with a μ-controller, LED, PD, and thermistor. Once the NFC reader supplies power to the device, the NFC chip rectifies the power and transfers to the μ-controller to drive the LEDs to emit light. The PD detects the backscattered light from the tissue and the thermistor obtains raw data corresponding to temperature. The collected data are transferred wirelessly to the NFC reader and processed to obtain the bio-signal (i.e., tissue oxygenation and temperature).

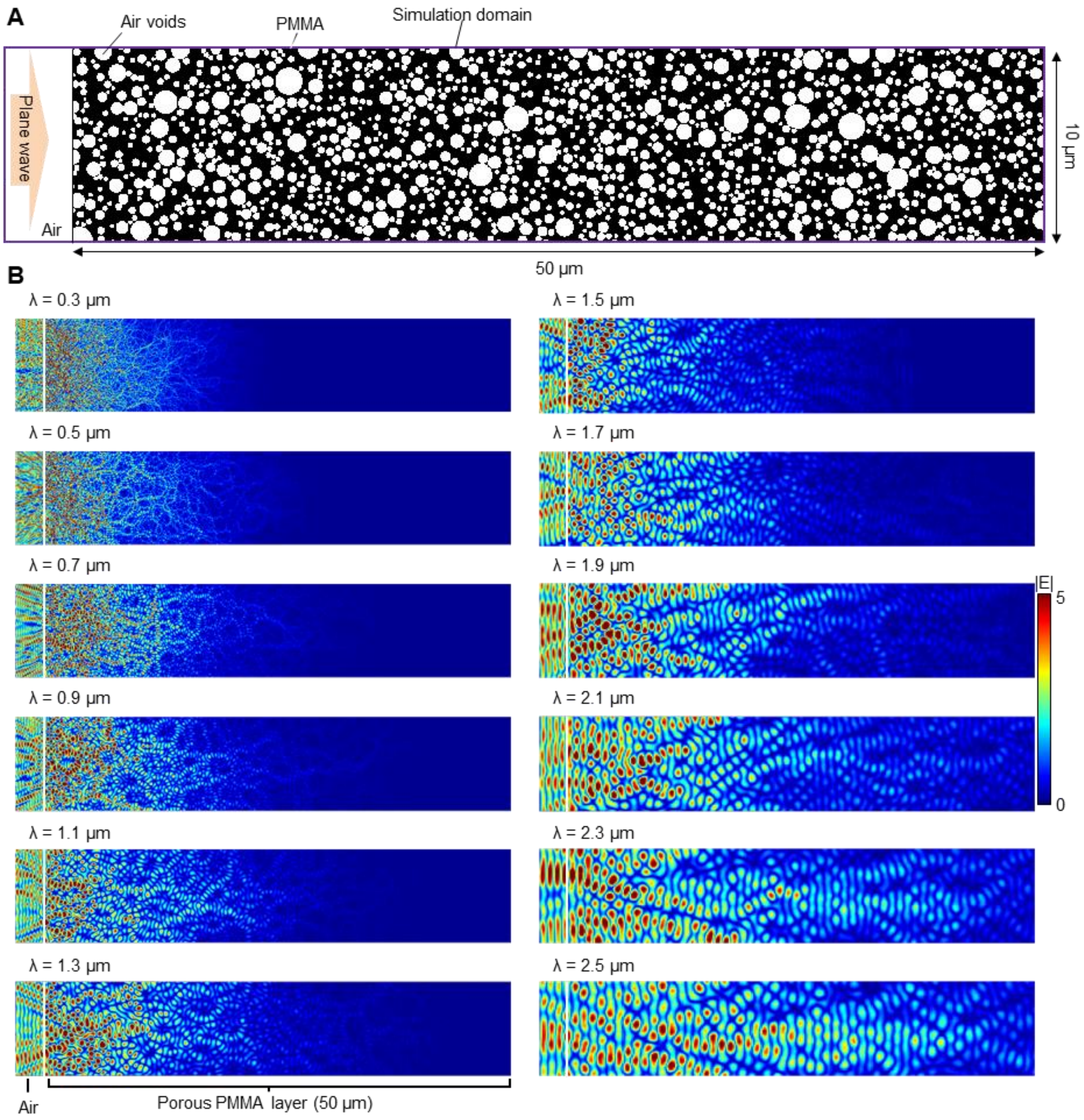

Figure S4. Electric field simulations on the computational model of *p*-PMMA with different wavelengths. (A) Simulation domain and randomized porous structure. The random void structure is generated by MATLAB using geometric parameters obtained from SEM images. (B) Electric field propagation simulations on *p*-PMMA with different wavelengths from 300 to 2500 nm, with a step of 200 nm. These results visualize the penetration depth of each photon into the *p*-PMMA layer. The overall *p*-PMMA layer thickness is 50 μm. Shorter wavelengths correspond to shallower penetration depths. In particular, the *p*-PMMA layer shows more effective blocking of wavelengths in the UV–VIS region.

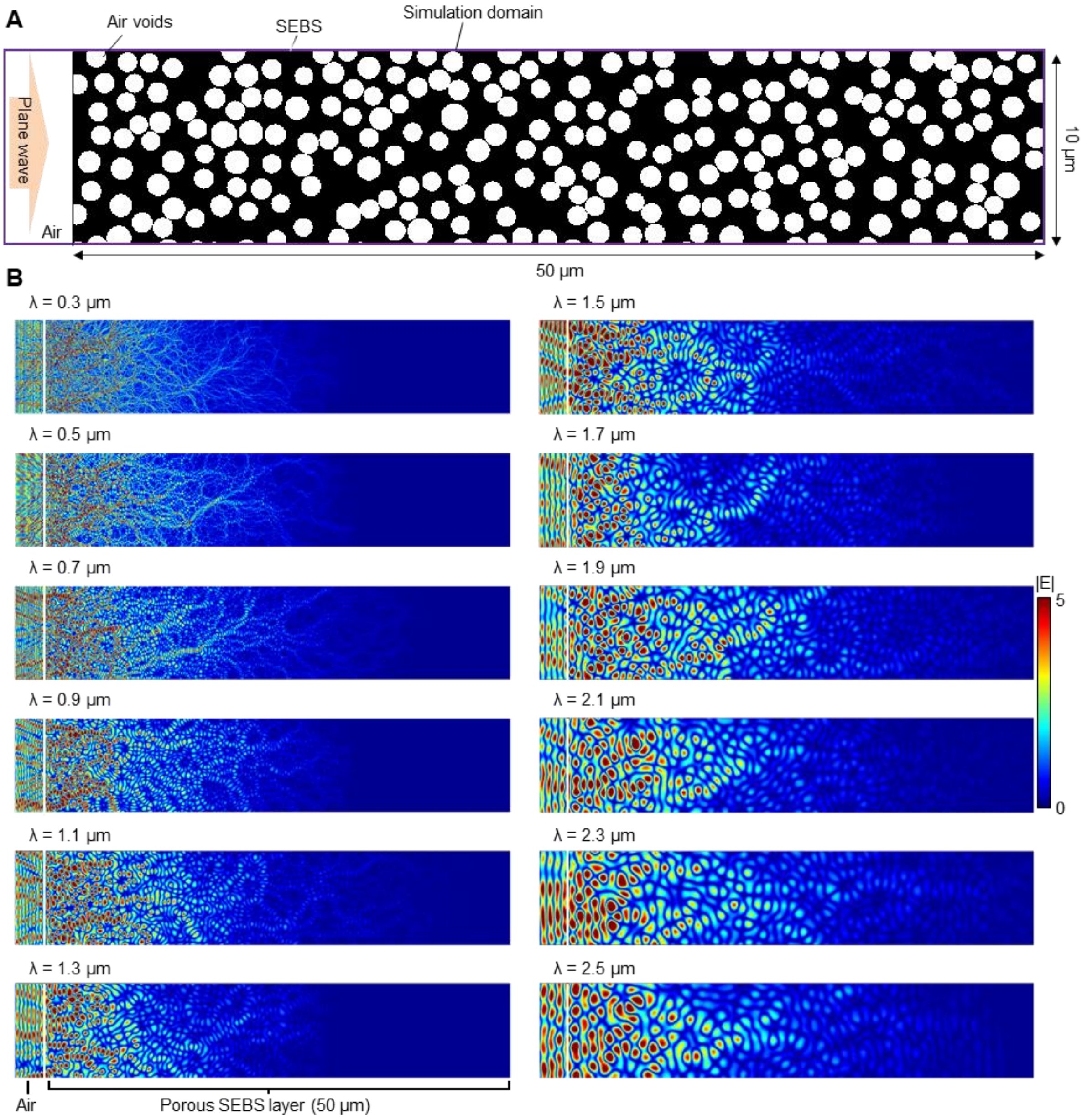

Figure S5. Electric field simulations on the computational model of *p*-SEBS with different wavelengths. (A) Simulation domain and randomized porous structure. The random void structure is generated by MATLAB using geometric parameters obtained from SEM images. (B) Electric field propagation simulations on *p*-SEBS with different wavelengths from 300 to 2500 nm, with a step of 200 nm. These results visualize the penetration depth of each photon into the *p*-SEBS layer. The overall *p*-SEBS layer thickness is 50 μm. Unlike the *p*-PMMA layer, the *p*-SEBS layer is suitable to shield longer wavelengths such as those in the NIR region.

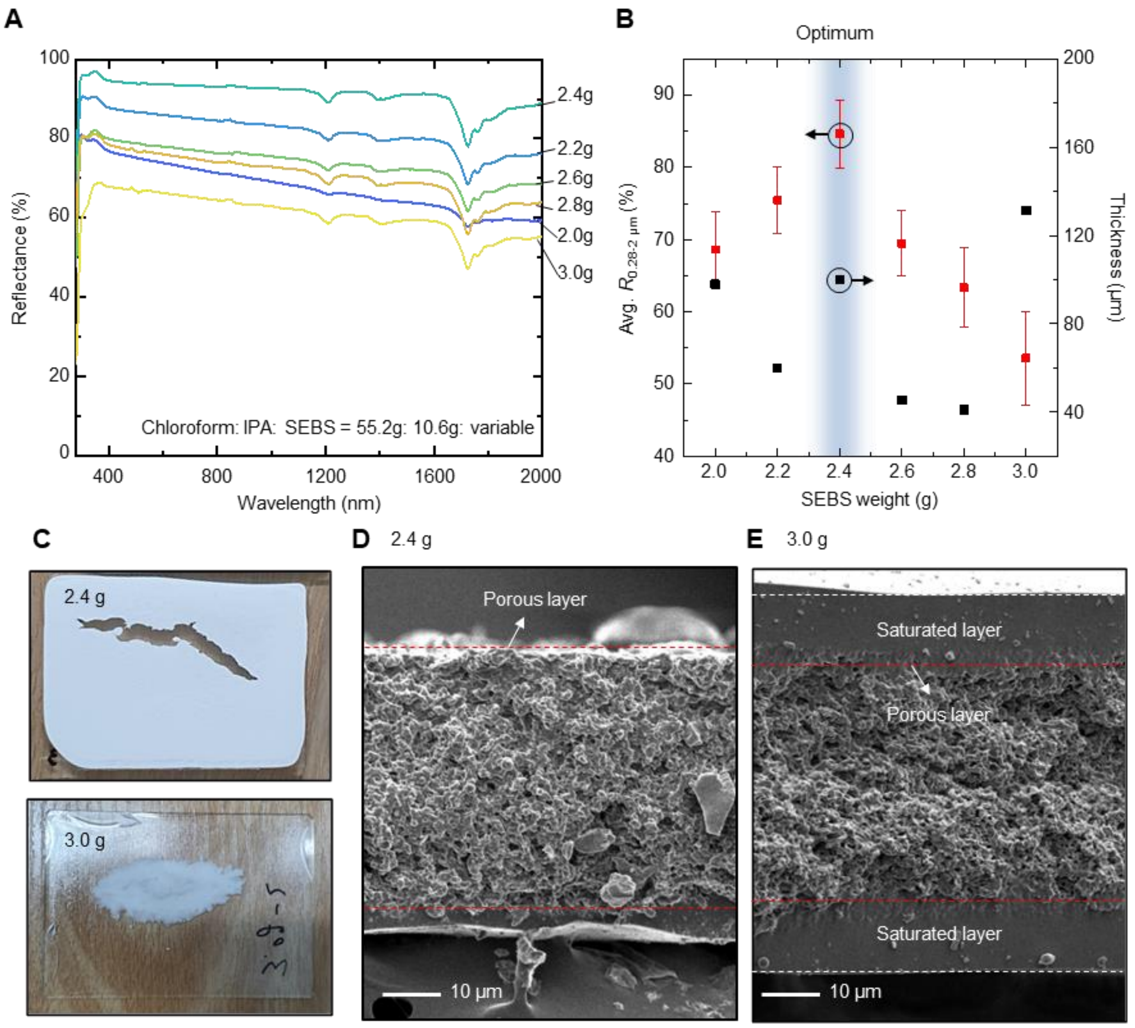

Figure S6. Optimization of *p*-SEBS in terms of weight ratio of solvent (chloroform), nonsolvent (IPA), and polymer (SEBS). (A) Reflectance spectra of *p*-SEBS depending on different SEBS weights from 2.0 to 3.0 g. The weights of chloroform and IPA are 55.2 and 10.6 g, respectively. (B) Summary of *p*-SEBSs with different SEBS weights with respect to the average reflectance from the wavelengths of 280 to 2000 nm and the layer thickness. The optimum *p*-SEBS was selected as that with the SEBS weight of 2.4 g. (C) Optical images of best and worst cases of *p*-SEBS (*i.e.,* 2.4 and 3.0 g). *p*-SEBS with the SEBS weight of 3.0 g appears transparent in most areas, whereas the *p*-SEBS with the SEBS weight of 2.4 g displays remarkably white surfaces, except for a small area. (D,E) SEM images of the *p*-SEBSs with SEBS weights of 2.4 and 3.0 g, respectively. The overall layer of the optimum *p*-SEBS is composed of a porous structure, whereas the *p*-SEBS with the SEBS weight of 3.0 g has saturated layers on the top and bottom sides. The *p*-SEBS with the SEBS weight of 3.0 g is the thickest sample, but this is due to the saturated layers, which are not helpful to enhance reflectance. This means that excessive doping of polymer hinders highly porous structures.

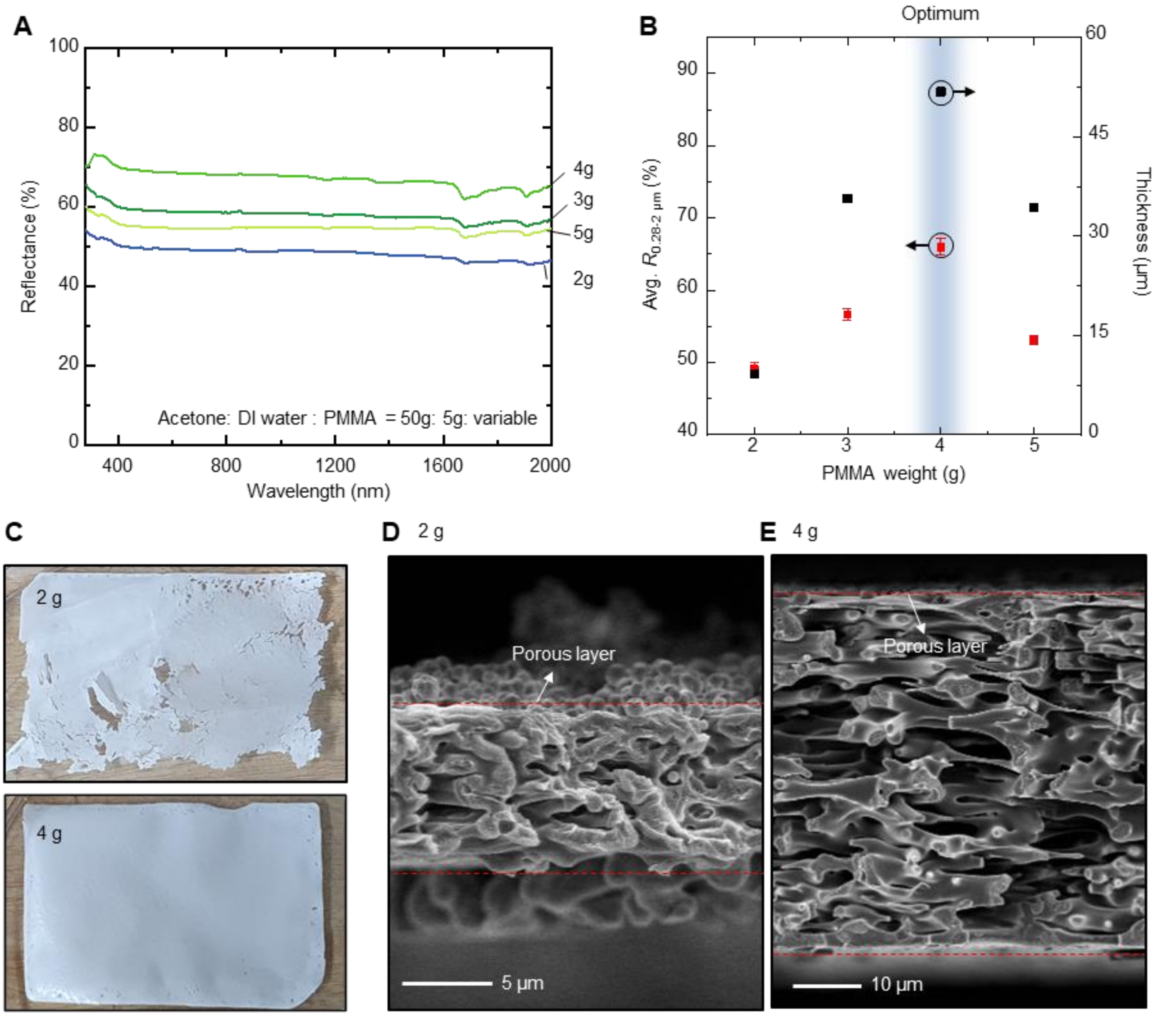

Figure S7. Optimization for porous *p*-PMMA in terms of weight ratio of solvent (acetone), nonsolvent (DI water), and PMMA (polymer). (A) Reflectance spectra of *p*-PMMA depending on different PMMA weights from 2.0 to 5.0 g. The weights of acetone and DI water are 50 and 5 g, respectively. (B) Summary of *p*-PMMAs with different PMMA weights with respect to the average reflectance from the wavelengths of 280 to 2000 nm and the layer thickness. The optimum *p*-PMMA was selected as that with the PMMA weight of 4 g. Unlike the *p*-SEBS case, in the *p*-PMMAs, the thicker sample exhibited the higher reflectance. However, the high doping of polymer cannot lead to thicker porous layers. The *p*-PMMA with the PMMA weight of 4 g is the optimum. (C) Optical images of best and worst cases of *p*-PMMA (*i.e.,* 2 and 4 g). The *p*-PMMA with the PMMA weight of 2 g was not densely formed, because the polymer chains were insufficient to fully connect with each other. In contrast, the *p*-PMMA with the PMMA weight of 4 g shows a white and dense layer. (D, E) SEM images of the *p*-PMMAs with the PMMA weights of 2 and 4 g, respectively. In the *p*-PMMA with the PMMA weight of 2 g, the formed porous layer is too thin (~4 μm), which is mechanically very weak. These results confirm that the *p*-PMMA with the PMMA weight of 4 g is the optimum.

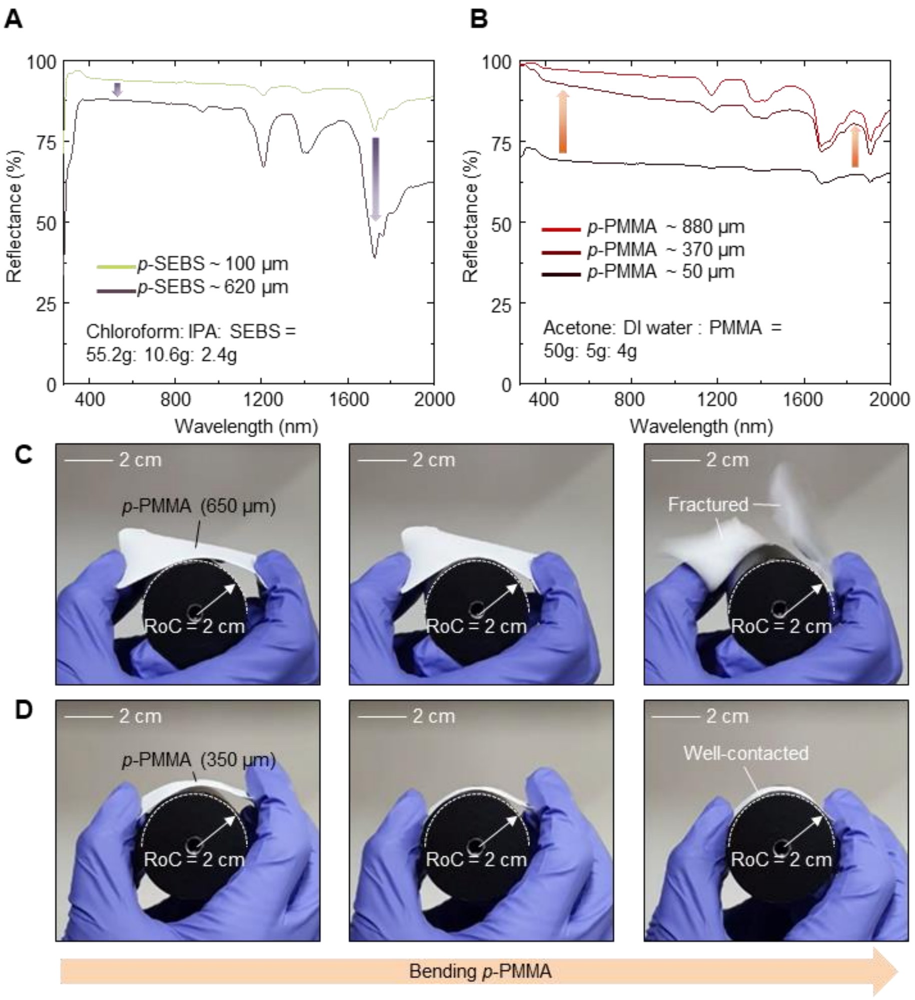

Figure S8. Measured reflectance spectra and flexibility of *p*-SEBS and *p*-PMMA depending on layer thickness. The weight ratio of solvents, non-solvents, and polymers are listed in the graphs. (A) Reflectance spectra of *p*-SEBSs with the thicknesses of 100 and 620 µm from the wavelengths of 280 to 2000 nm. Thicker *p*-SEBSs show lower reflectance than that of 100-µm-thick *p*-SEBS, particularly in the NIR region. (B) Reflectance spectra of *p*-PMMAs with the thicknesses of 50, 370, and 880 µm from the wavelengths of 280 to 2000 nm. Thicker *p*-PMMAs show enhanced reflectance in the UV and VIS ranges; however, the improvement in NIR reflectance is insufficient. The thicker samples were fabricated by spraying mixtures of solvent, non-solvent, and polymer on the optimized *p*-SEBS (*i.e., p*-SEBS with thickness ~100 µm) and *p*-PMMA (*i.e., p*-PMMA with thickness ~50 µm), as depicted in Figure S5 and S6. These results provide the design rule of NMVP to increase the reflectance in both the UV–VIS and NIR regions, requiring the integration of *p*-SEBS, which serves as a balanced reflector in all ranges, and *p*-PMMA, which is helpful to more strongly reject the UV–VIS range. (C, D) Bending tests on thicker and thinner *p*-PMMAs such as (C) 650 µm and (D) 350 µm, respectively. The tested radius of curvature (RoC) is 2 cm. The thicker *p*-PMMA is broken when the sample is bent for fitting with RoC of 2 cm.

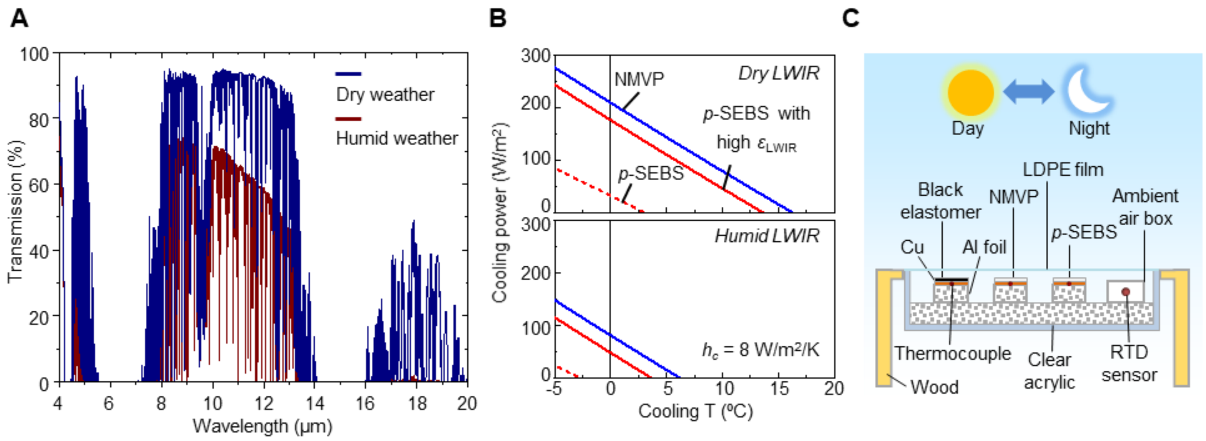

Figure S9. Sky transmission spectra, expected cooling performances, and measurement setup. (A) Calculated sky transmission spectra for dry and humid weather using MODTRAN 6. The dry and humid weather conditions are based on an area at mid-latitude during winter and summer seasons. (B) Calculated cooling power and temperature for NMVP, *p*-SEBS, and *p*-SEBS with high emissivity in LWIR (ε_{LWIR}) under clear and humid LWIR windows. Non-radiative heat-exchange coefficient is set to 8 W/m^2/K. (C) Schematic illustration of measurement setup.

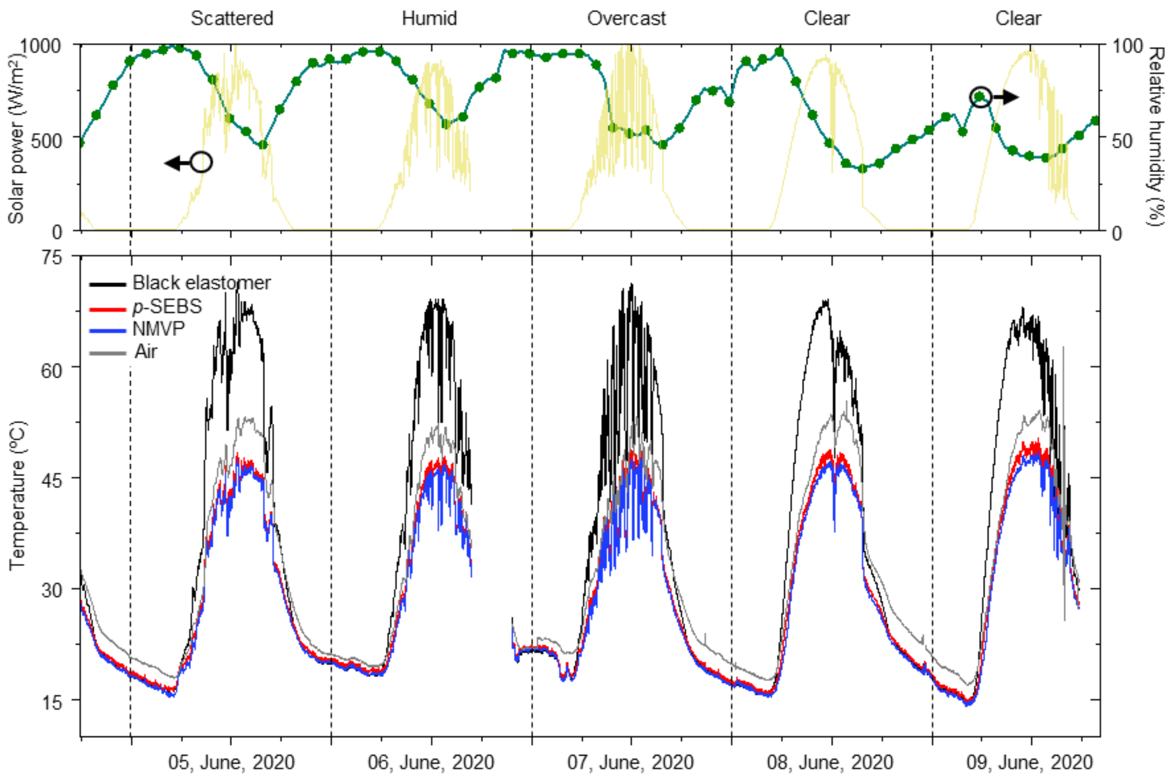

Figure S10. Five-day continuous cooling measurement with various climates. Temperature measurements for black elastomer, *p*-SEBS, and NMVP samples and ambient air inside the setup, as well as solar power and relative humidity measurements. On all days, NMVP exhibits sub-ambient cooling performance.

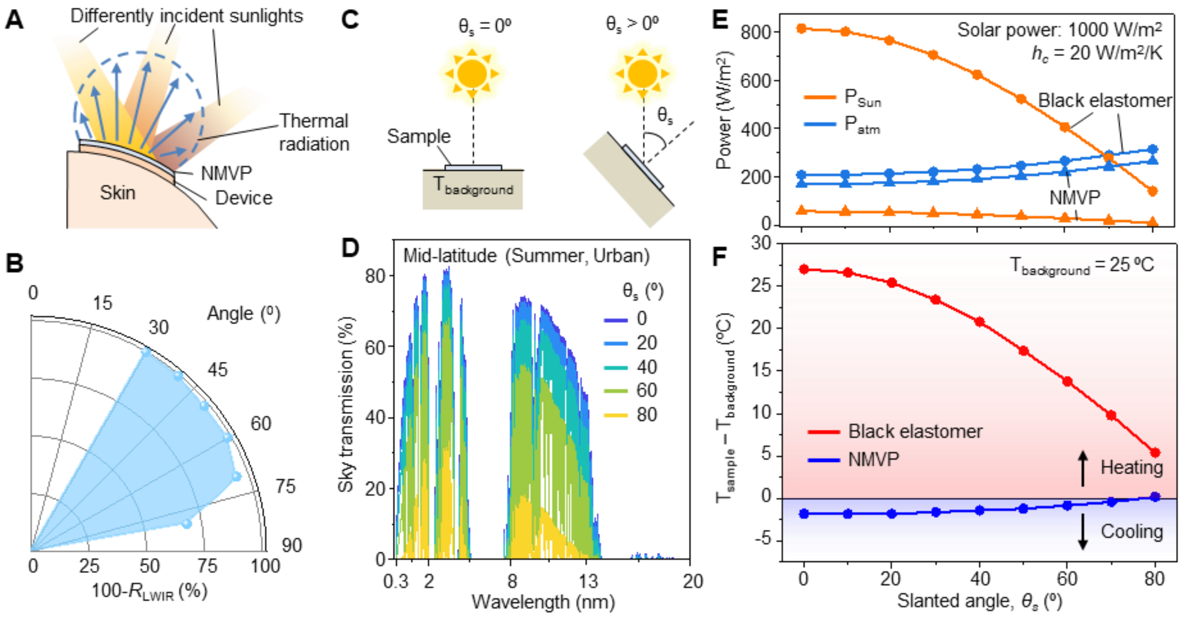

Figure S11. Angular cooling features of NMVP. (A) Schematic of NMVP-integrated device on skin. Differently incident solar light and diverse thermal radiation are considered for the use of wearable electronics. (B) Measured angular response of NMVP for emissivity in LWIR range. (C) Schematic of tilted case of NMVP at slanted angle, θ_s. $T_{\text{background}}$ is 25 °C. The solar insolation is reduced in proportion to the increasing cosine of θ_s. (D) LWIR atmospheric window transmission variation depending on θ_s. Tilting the sample degrades the cooling performance owing to increasing LWIR opacity. (E, F) Calculated radiative powers of P_{Sun} and P_{atm} (E) and $T_{sample}–T_{background}$ (F) as a function of θ_s. At oblique angles, P_{sun} is remarkably reduced in black elastomer, but the sample is always hotter than the background. In contrast, NMVP always maintains cooling status, except for θ_s = 80°, owing to the near cancellation of reduced P_{Sun} and increased P_{atm}.

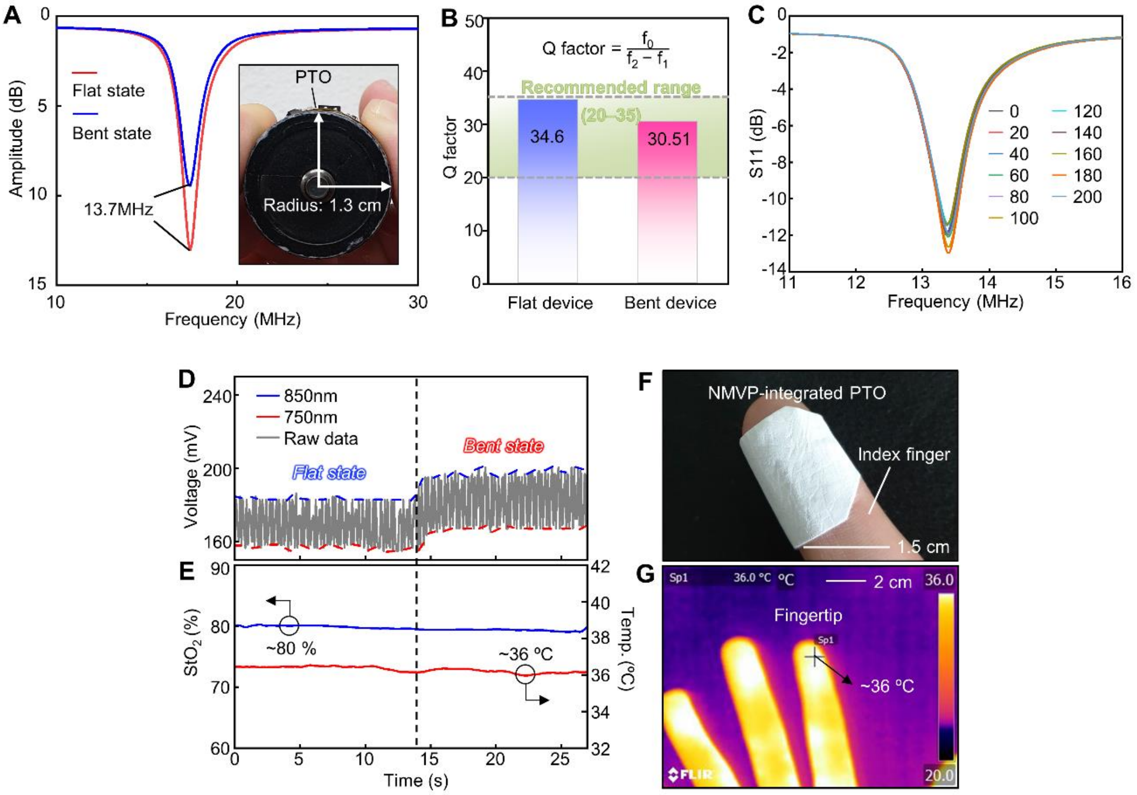

Figure S12. Bending test result of PTO. (A) Network analyzer measurement result of device in flat geometry and in bent geometry with radius of curvature (RoC) of 1.3 cm. (B) Q factors of flat device and bent device. The frequency characteristics of the device barely degrade with geometric changes and remain in the recommended range. The recommended range for Q factor is from 20 to 35. As the Q factor is high, the coil may be selective, which can result in narrow bandwidth of the resonance and also affect the NFC signal.[10, 11] (C) Measured frequency characteristics of PTO for every 20 bending cycles. (D) Raw data, (E) StO_2 (blue) and temperature (red) measured by PTO at fingertip in flat and bent states. (F) Image of NMVP-integrated PTO with index finger. (G) Thermal image of fingers with temperature of ~36 °C. Since more tight contact with the skin increases the total amount of penetrated light to the skin, the raw data are slightly increased in a bent state. StO_2 and temperature results measured in the bent state barely changed compared to the flat state (~80 % and ~36 °C). The temperature results measured by NMVP-integrated PTO are similar to the result of the thermal image.

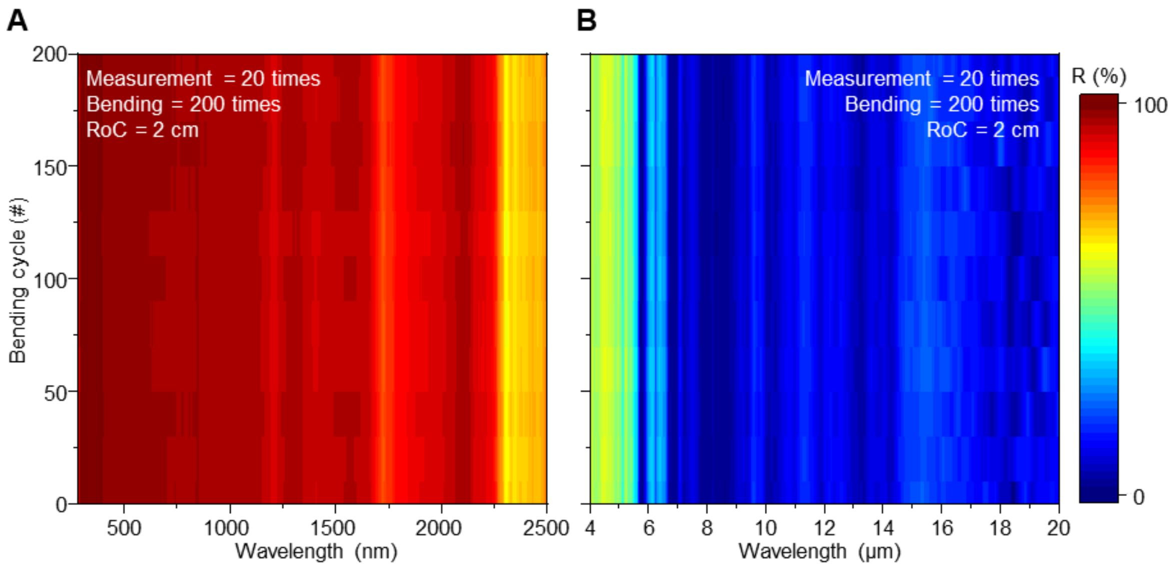

Figure S13. Reflectivity spectra in UV-VIS-NIR region and FIR region. Measured reflectivity spectra of NMVP in (A) UV-VIS-NIR and (B) FIR region per 20 folding.

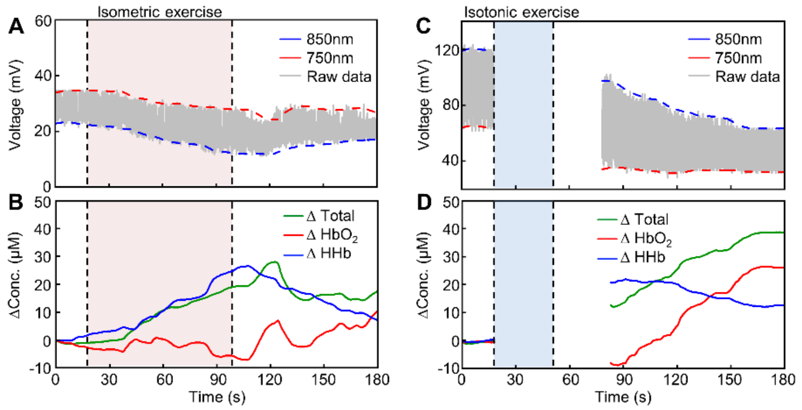

Figure S14. Exercise experiment. (A, B) Isometric exercise experiment results of (A) raw data and (B) hemoglobin concentration. The red area indicates the exercise period. (C, D) Isotonic exercise experiment results of (C) raw data and (D) hemoglobin concentration. The blue area indicates exercise period.

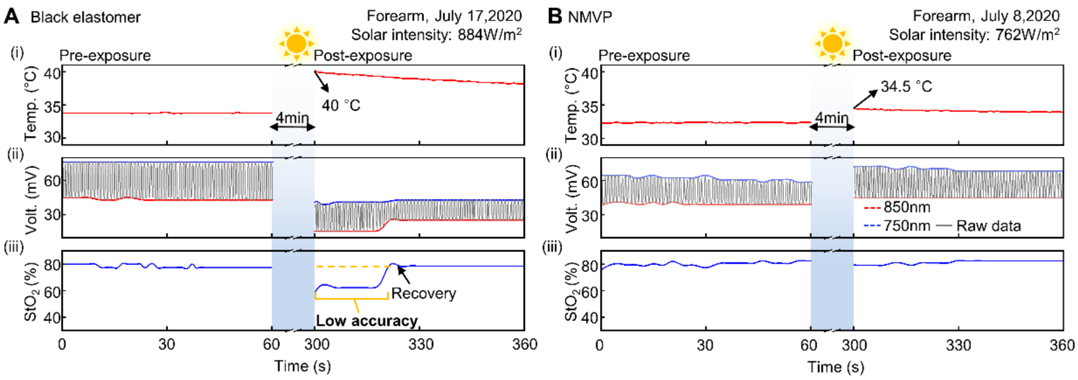

Figure S15. Comparison experiment with black elastomer and NMVP implemented in summer. Comparison of measurements pre-exposure and post-exposure with (A) BE-integrated device and (B) NMVP-integrated device attached to forearm. Sky-blue regions represent time period (4 min) of direct sunlight exposure.

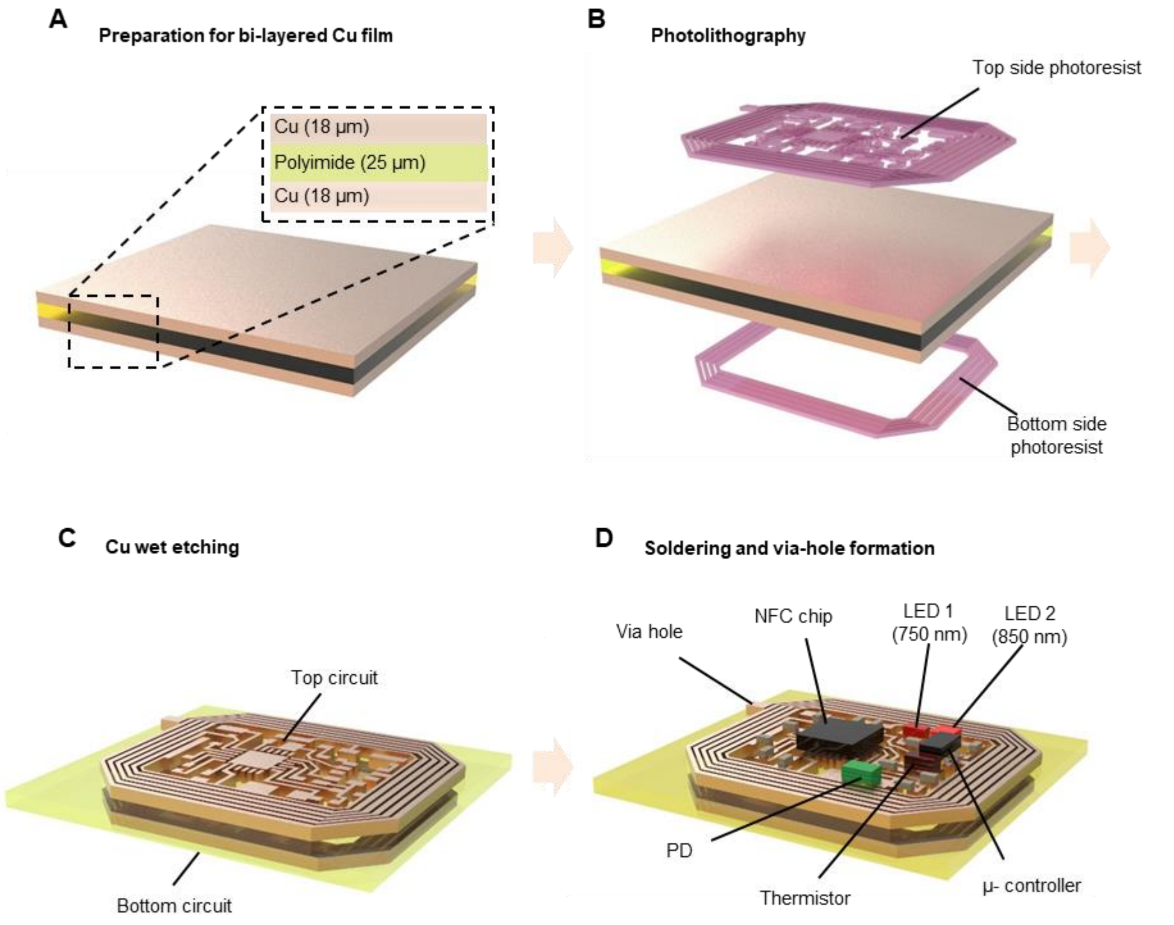

Figure S16. Tissue oximeter fabrication process. (A) Cu (18 μm)/ polyimide (25 μm)/ Cu (18 μm) foil. (B) Exploded view of the foil with patterned photoresist. The photolithography process was performed with AZ9260 photoresist on both the top and bottom sides. (C) Cu wet etching to pattern the circuit with CE-100 Cu etchant for 8 min. (D) Soldering and via-hole formation using silver paste.

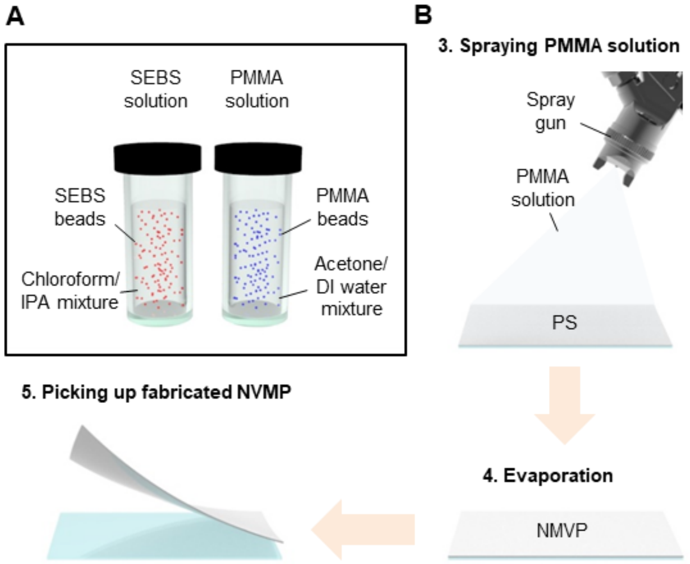

Figure S17. NMVP fabrication process. (A) Dissolving SEBS and PMMA beads in each solvent/nonsolvent mixture. (B) The fabrication of NMVP begins with drop-casting SEBS on glass slide. Then, the casted SEBS solution is evaporated to obtain dried *p*-SEBS film. Next, the dissolved PMMA solution is sprayed on the *p*-SEBS layer. The sprayed PMMA solution is evaporated for the formation of the NMVP layer. Finally, the fabricated NMVP is picked up using tweezers.

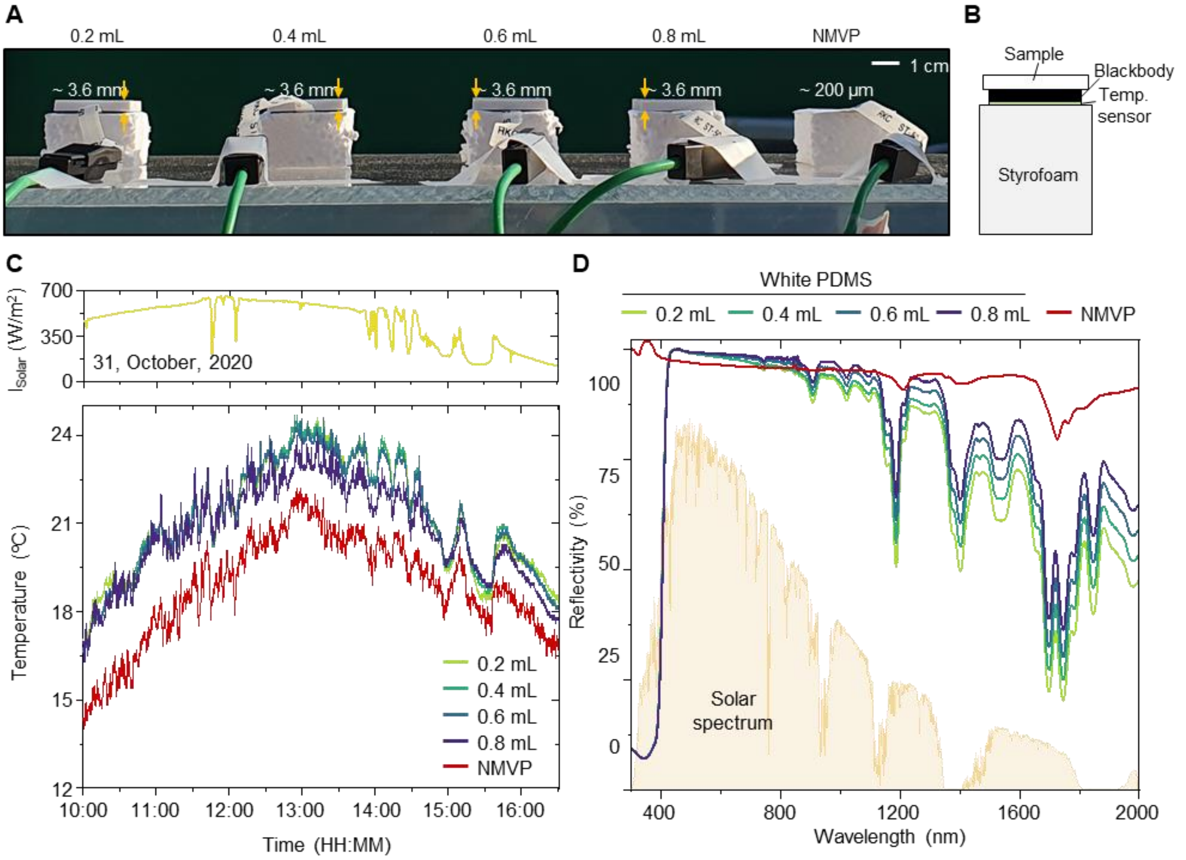

Figure S18. (A) Photograph of one NMVP and four white PDMSs with dye densities of 0.2, 0.4, 0.6, and 0.8 mL. The PDMS weights were 11 g, with a 10:1 ratio of base elastomer and curing agent. These PDMSs have a sufficiently thick layer, ~ 3.6 mm. The NMVP shows a very thin layer, ~ 0.2 mm, compared to those of PDMSs. (B) Schematic illustration for individual temperature measurement unit composed of the sample, blackbody, temperature sensor, and Styrofoam. The sample are white PDMSs and NMVP. The blackbody is a commercial broadband absorber (Acktar Light Absorbent Foil; Metal Velvet Adhesive Foil Sheet, Edmund Optics, USA) with the absorptivity of 99.9% from the wavelength of 0.3 to 13.3 μm. A temperature sensor below the blackbody records its temperature. The Styrofoam works as a thermal insulation layer at the bottom.

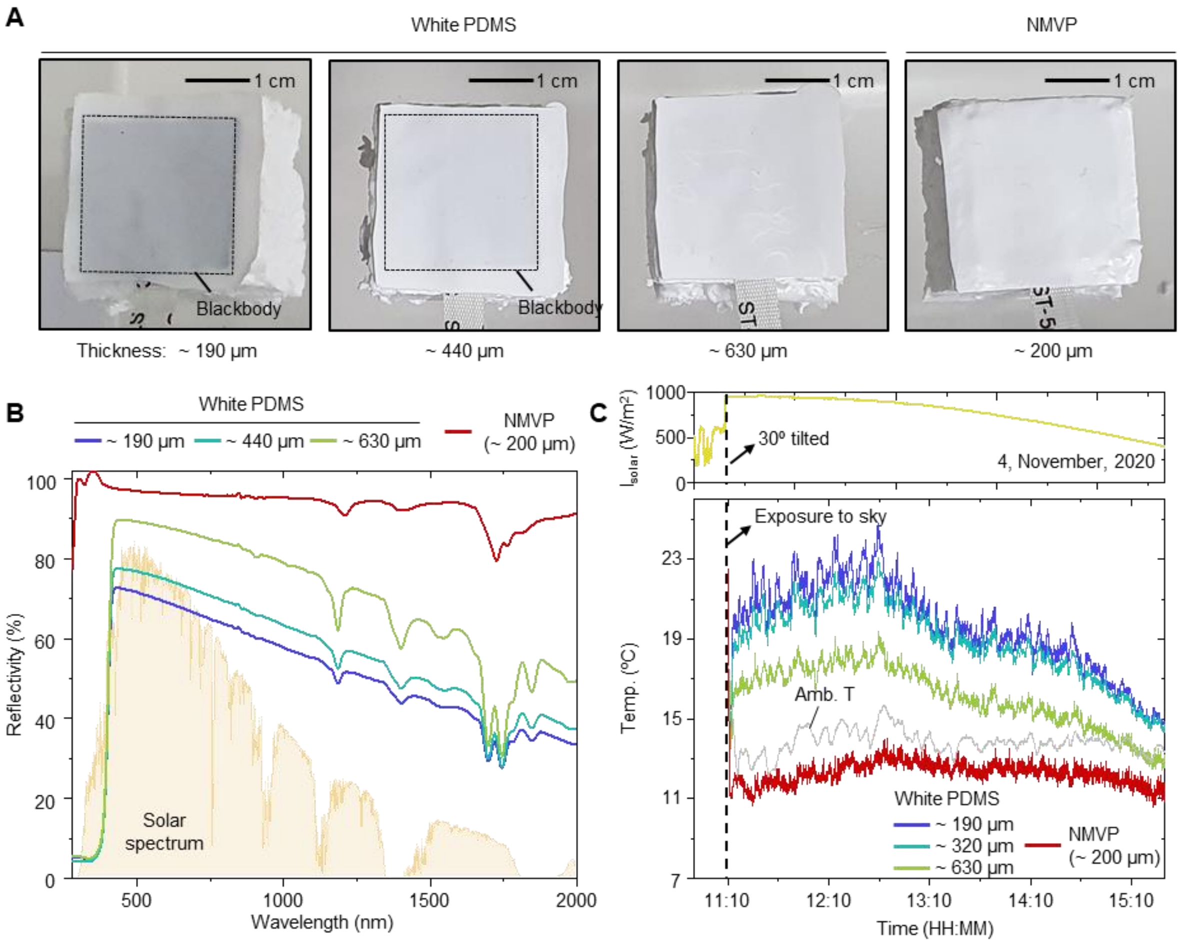

Figure S19. (A) Photographs of three white PDMSs with different thicknesses of ~ 190, 440, and 630 μm and NMVP with a thickness of ~ 200 μm. (B) Reflectance spectra of three white PDMSs with thicknesses of ~ 190, 440, and 630 μm and NMVP with a thickness of ~ 200 μm . (C) Measured (top) solar intensity and (bottom) temperatures of white PDMSs, NMVP, and ambient air. We tilted the samples and solar sensor at 30° to measure the temperature under st rong sunlight (the measurement was performed in late autumn on November 4, 2020). Before tilting, the solar intensity is remarkably weak, ~ 600 W/m^2. 'Amb. T' denotes ambient air tem perature.

Reference	Operating system	Sensor (type)	Thermal management method	Outdoor usable	Cooling Performance	Notes
**This work**	**Wireless/Battery -free (NFC)**	**Optoelectronics**	**Radiative cooling**	**O**	**~7°C cooling compared ambient**	**R_{solar} = ~96% / ε_{LWIR} = ~ 90%, Nonmetallic**
[6]	Wireless/Battery-free (NFC)	Optoelectronics	Non-thermal management	X	N/A	Solar absorbing material (black encapsulation)
[7]	Wireless/Battery-free (NFC)	Optoelectronics	Non-thermal management	X	N/A	Solar absorbing material (black encapsulation)
[12]	Wireless/Battery-free (NFC)	Optoelectronics	Non-thermal management	X	N/A	Solar absorbing material (Cu and skin)
[13]	Wireless (Bluetooth)	Optoelectronics	Non-thermal management	X	N/A	Solar absorbing material (Black encapsulation)
[14]	Wireless (Bluetooth)	Non-optoelectronics	Non-thermal management	X	N/A	Solar absorbing material (Cu and skin) Battery required
[15]	Wired	Optoelectronics	Thin metallic heat sink	X	48°C to 39°C	Solar absorbing material (Cu), Metallic
[16]	Wired	Non-optoelectronics	Phase-shift material/ heat sink layer	X	130°C to 50°C (too high temperature for wearables)	Solar absorbing material (Cu), Metallic
[17]	Wireless (Bluetooth)	Optoelectronics	Radiative cooling	△	N/A	Battery required
[18]	Wired	Non-optoelectronics	Radiative cooling	O	~6°C cooling compared bare skin	Low cooling performance (R_{solar} = ~90% / ε_{LWIR} = N/A), **Nonmetallic**

Table S1. Specifications of previous reported wearable sensors in terms of operating system, sensing type, thermal management method, outdoor usability, cooling performance, and remarks. Our proposed layout only deals with wireless/battery-free optoelectronics with outdoor usability. Orange, green, and blue font are used to describe wireless/battery-free operating system, optoelectronics sensor, and the thermal management method, respectively. Our device satisfies all these aspects.

Reference	Matrix	Scatters	Main applications	Cooling performance	Notes
**This work**	**PMMA/SEBS**	**Air**	**Outdoor-useable tissue oximeter**	**~7°C cooling compared ambient**	R_{solar} = ~96% / ε_{LWIR} = ~ 90%
[18]	SEBS	Air	Heat management of ECG/EMG sensor	~6°C cooling compared bare skin	R_{solar} = ~90% / ε_{LWIR} = ~ 85%
[19]	P(VdF-HFP)	Air	Paintable radiative cooler	~6°C cooling compared ambient air	R_{solar} = ~96% / ε_{LWIR} = ~ 97%
[20]	Cellulose	Air	Radiative cooling wood	~4°C cooling compared ambient air	R_{solar} = ~96% / ε_{LWIR} = ~ 90%
[21]	P(VdF-HFP) or PE	Air or alcohol	Optical and thermal regulation	~ 3.2°C and ~ 21.4 °C cooling and heating compared ambient air	ΔT_{solar} = ~ 0.74 (Cooling to heating) ΔT_{solar} = ~ 0.33 / ΔT_{LWIR} = ~ -0.64 (Icehouse to greenhouse)
[22]	PDMS	Air	Heating/cooling mode conversion	~ 5°C and ~ 18°C cooling and heating compared ambient air	R_{solar} (ε_{solar}) = ~ 93% (95%) / ε_{LWIR} = ~ 94%
[23]	PAN	Air	Solar opaque and IR transparent film	Near-ambient	R_{solar} = ~95% / T_{LWIR} = ~ 70%,
[24]	PDMS	Al_2O_3	Bio-inspired flexible cooling film	~ 5.1°C cooling compared ambient air	R_{solar} = ~95% / ε_{LWIR} = ~ 96%,
[25]	P(VdF-HFP)	Air and colored dyes	Less heating color film	~ 3.0 to 7.3°C cooling compared commercial paints	R_{solar} = ~ 40 to 72% / ε_{LWIR} = ~ 90%

Table S2. Recently-developed non-metallic radiative coolers for various applications. Our radiative cooler demonstrates the exceptional radiative cooling performance and the applicability for wearable optoelectronics.

Layer	Thickness (mm)	Wavelength	Absorption coefficient (cm^{-1})	Scattering coefficient (cm^{-1})	Anisotropic factor	Refractive index
Epidermis	0.1	750	2	190	0.8	1.4
		850	1.3	176	0.8	1.4
Dermis	2	750	1.35	118	0.8	1.4
		850	1.04	106.5	0.8	1.4

Table S3. The optical characteristics and thickness of epidermis and dermis considered for simulation.[26, 27]

Reference

[1] H. Zhang, P. Gutruf, K. Meacham, M. C. Montana, X. Zhao, A. M. Chiarelli, A. Vázquez□Guardado, A. Norris, L. Lu, Q. Guo, C. Xu, Y. Wu, H. Zhao, X. Ning, W. Bai, I. Kandela, C. R. Haney, D. Chanda, R. W. Gereau, J. A. Rogers, *Sci. Adv.* **2019**, *5*, eaaw0873.
[2] F. Scholkmann, M. Wolf, *J. Biomed. Opt.* **2013**, *18*, 10.
[3] W. G. Zijlstra, A. Buursma, W. P. Meeuwsen-van der Roest, *Clin. Chem.* **1991**, *37*, 9.
[4] C. W. Sun, C. C. Chuang, *Hemodynamics-New Diagnostic and Therapeutic Approaches*, **2012**, *3*.
[5] T. Van Vo, P. E. Hammer, M. L. Hoimes, S. Nadgir, S. Fantini, *IEEE Trans. Biomed. Eng.* **2007**, 54, 4
[6] J. Kim, G. A. Salvatore, H. Araki, A. M. Chiarelli, Z. Xie, A. Banks, X. Sheng, Y. Liu, J. W. Lee, K.□I. Jang, S. Y. Heo, K. Cho, H. Luo, B. Zimmerman, J. Kim, L. Yan, X. Feng, S. Xu, M. Fabiani, G. Gratton, Y. Huang, U. Paik, J. A. Rogers, *Sci. Adv.* **2016**, *2*, e1600418.
[7] J. Kim, P. Gutruf, A. M. Chiarelli, S. Y. Heo, K. Cho, Z. Xie, A. Banks, S. Han, K.-I. Jang, J. W. Lee, K.□T. Lee, X. Feng, Y. Huang, M. Fabiani, G. Gratton, U. Paik, J. A. Rogers, *Adv. Funct. Mater.* **2017**, *27*, 1604373.
[8] E. V. Salomatina, B. Jiang, J. Novak, A. N. Yaroslavsky, *J. Biomed. Opt.* **2006,** *11*, 064026
[9] S. Wang, J. Zhao, H. Lui, Q. He, H. Zeng, *J. Photochem. Photobiol. B-Biol.* **2011**, *105*, 183.
[10] NXP, PN7120 Antenna design and Matching Guide, **2016**, Rev. 1.1.
[11] A. Lazaro, R. Villarino, D. Girbau, Sensors **2018**, *18*, 11.
[12] H. Jeong, L. Wang, T. Ha, R. Mitbander, X. Yang, Z. Dai, S. Qiao, L. Shen, N. Sun, N. Lu, *Adv. Mater. Technol.* **2019**, *4*, 1900117
[13] K.-I. Jang, S. Y. Han, S. Xu, K. E. Mathewson, Y. Zhang, J.-W. Jeong, G.-T. Kim, R. C. Webb, J. W. Lee, T. J. Dawidczyk, R. H. Kim, Y. M. Song, W.-H. Yeo, S. Kim, H. Cheng, S. I. Rhee, J. Chung, B. Kim, H. U. Chung, D. Lee, Y. Yang, M. Cho, J. G. Gaspar, R. Carbonari, M. Fabiani, G. Gratton, Y. Huang, J. A. Rogers, *Nat. Commun.* **2014**, *5*, 4779.
[14] H. Kim, Y. Kim, M. Mahmood, S. Kwon, N. Zavanelli, H. Kim, F. Epps, Y. Rim, and W. H. Yeo, *Adv. Sci.* **2020**, *7*, 2000810.
[15] H. H. Jung, J. Song, S. Nie, H. N. Jung, M. S. Kim, J.-W. Jeong, Y. M. Song, J. Song, K.-I. Jang, *Adv. Mater. Technol.* **2018**, *3*, 1800159.
[16] Y. Shi, C. Wang, Y. Yin, Y. Li, Y. Xing, J. Song, *Adv. Funct. Mater.* **2019**, *29*, 1905470.
[17] X. Hu, M. Tian, T. Xu, X. Sun, B. Sun, C. Sun, X. Liu, X. Zhang, L. Qu, *ACS Nano* **2020**, *14*, 559.
[18] Y. Xu, B. Sun, Y. Ling, Q. Fei, Z. Chen, X. Li, P. Guo, N. Jeon, S. Goswami, Y. Liao, S. Ding, Q. Yu, J. Lin, G. Huang, Z. Yan, *Proc. Natl. Acad. Sci. U.S.A* **2020**, *117*, 205.
[19] J. Mandal, Y. Fu, A. C. Overvig, M. Jia, K. Sun, N. N. Shi, H. Zhou, X. Xiao, N. Yu, Y. Yang, *Science* **2018**, *362*, 315.
[20] T. Li, Y. Zhai, S. He, W. Gan, Z. Wei, M. Heidarinejad, D. Dalgo, R. Mi, X. Zhao, J. Song, J. Dai, C. Chen, A. Aili, A. Vellore, A. Martini, R. Yang, J. Srebric, X. Yin, L. Hu, *Science* **2019**, *364*, 760.
[21] J. Mandal, M. Jia, A. Overvig, Y. Fu, E. Che, N. Yu, Y. Yang, *Joule* **2019**, *3*, 3088.
[22] H. Zhao, Q. Sun, J. Zhou, X. Deng, J. Cui, *Adv. Mater.* **2020**, *32*, 2000870.
[23] H. Kim, S. McSherry, B. Brown, A. Lenert, *ACS Appl. Mater. Interfaces* **2020**, *12*, 43553.
[24] H. Zhang, K. C. S. Ly, X. Liu, Z. Chen, M. Yan, Z. Wu, X. Wang, Y. Zheng, H. Zhou, T. Fan, *Proc. Natl. Acad. Sci. U.S.A* **2020**, *117*, 14657..

[25] Y. Chen, J. Mandal, W. Li, A. Smith-Washington, C.-C. Tsai, W. Huang, S. Shrestha, N. Yu, R. P. S. Han, A. Cao, Y. Yang, *Sci. Adv.* **2020**, *6*, eaaz5413.
[26] E. V. Salomatina, B. Jiang, J. Novak, A. N. Yaroslavsky, *J. Biomed. Opt.* **2006,** *11*, 064026
[27] S. Wang, J. Zhao, H. Lui, Q. He, H. Zeng, *J. Photochem. Photobiol. B-Biol.* **2011**, *105*, 183.
